# Impact of SARS-CoV-2 infective exacerbation of chronic obstructive pulmonary disease on clinical outcomes in a prospective cohort study of hospitalised adults

**DOI:** 10.1177/01410768231184162

**Published:** 2023-07-05

**Authors:** Catherine Hyams, George Qian, George Nava, Robert Challen, Elizabeth Begier, Jo Southern, Maria Lahuerta, Jennifer L Nguyen, Jade King, Anna Morley, Madeleine Clout, Nick Maskell, Luis Jodar, Jennifer Oliver, Gillian Ellsbury, John M McLaughlin, Bradford D Gessner, Adam Finn, Leon Danon, James W Dodd, The Avon CAP Research Group

**Affiliations:** 1 Academic Respiratory Unit and Bristol Vaccine Centre, 1980 University of Bristol, Bristol, BS15, UK; 2 Engineering Mathematics, 1980 University of Bristol, Bristol, Bristol, BS8, UK; 3 Academic Respiratory Unit, University of Bristol, Southmead Hospital, Bristol, Bristol, BS15, UK; 4 Vaccines Medical Development, Scientific and Clinical Affairs, Pfizer Inc., Collegeville, PA 19426, USA; 5 Clinical Research and Imaging Centre, UHBW NHS Trust, Bristol, Bristol, BS2, UK; 6 Academic Respiratory Unit, Southmead Hospital, Bristol, Bristol, BS15, UK; 7 Bristol Vaccine Centre and Population Health Sciences, University of Bristol, Bristol, BS2, UK; 8 Vaccines Medical Affairs, Pfizer Ltd, Tadworth, KT20, UK; 9 Bristol Vaccine Centre, 1980 Cellular and Molecular Medicine and Population Health Sciences, University of Bristol, Bristol, BS2, UK; 10 Academic Respiratory Unit and Population Health Sciences, University of Bristol, Southmead Hospital, Bristol, BS15, UK

**Keywords:** COVID-19, SARS-CoV-2, acute exacerbation of COPD, COPD, airways disease

## Abstract

**Objectives:**

To determine whether acute exacerbations of chronic obstructive pulmonary disease (AECOPD) triggered by severe acute respiratory syndrome coronavirus 2 (SARS-CoV-2), have worse outcomes than AECOPD caused by other infectious agents or non-infective AECOPD (NI-COPD).

**Design:**

A two-hospital prospective cohort study of adults hospitalised with acute respiratory disease. We compared outcomes with AECOPD and a positive test for SARS-CoV-2 (n = 816), AECOPD triggered by other infections (n = 3038) and NI-COPD (n = 994). We used multivariable modelling to adjust for potential confounders and assessed variation by seasons associated with different SARS-CoV-2 variants.

**Setting:**

Bristol UK, August 2020–May 2022.

**Participants:**

Adults (≥18 y) hospitalised with AECOPD.

**Main outcome measures:**

We determined the risk of positive pressure support, longer hospital admission and mortality following hospitalisation with AECOPD due to non-SARS-CoV-2 infection compared with SARS-CoV-2 AECOPD and NI-COPD.

**Results:**

Patients with SARS-CoV-2 AECOPD, in comparison to non-SARS-CoV-2 infective AECOPD or NI-COPD, more frequently required positive pressure support (18.5% and 7.5% vs. 11.7%, respectively), longer hospital stays (median [interquartile range, IQR]: 7 [3–15] and 5 [2–10] vs. 4 [2–9] days, respectively) and had higher 30-day mortality (16.9% and 11.1% vs. 5.9%, respectively) (all *p* < 0.001). In adjusted analyses, SARS-CoV-2 AECOPD was associated with a 55% (95% confidence interval [95% CI]: 24–93), 26% (95% CI: 15–37) and 35% (95% CI: 10–65) increase in the risk of positive pressure support, hospitalisation length and 30-day mortality, respectively, relative to non-SARS-CoV-2 infective AECOPD. The difference in risk remained similar during periods of wild-type, Alpha and Delta SARS-CoV-2 strain dominance, but diminished during Omicron dominance.

**Conclusions:**

SARS-CoV-2-related AECOPD had worse patient outcomes compared with non-SARS-CoV-2 AECOPD or NI-AECOPD, although the difference in risks was less pronounced during Omicron dominance.

## Introduction

Individuals with chronic obstructive pulmonary disease (COPD) are susceptible to exacerbations, defined as an acute worsening of respiratory symptoms from a patient’s stable state.^1^ Acute exacerbations of chronic obstructive pulmonary disease (AECOPD) lead to increased healthcare utilisation, reduced health-related quality of life and accelerated loss of lung function, with severe exacerbations increasing the risk of death.^2^ Viral infections are the commonest cause of AECOPD, though there can also be bacterial, non-infective or combined triggers.^3^ Viral AECOPD cause more prolonged symptoms and longer hospital stays^4^ than non-viral exacerbations. Data from a longitudinal observational studies of COPD patients and age-matched controls showed that COPD patients have an increased frequency of hospitalisation despite a similar frequency of respiratory viral infection.^5^ COPD is a risk factor for hospitalisation during viral epidemics, including influenza^6^ and respiratory syncytial virus.^7^ The underlying mechanisms remain unclear. In both mouse models and human studies of COPD patients, respiratory viruses induce impaired antiviral or innate responses in COPD patients.^8,9^ Impaired immune responses to viral pathogens may contribute to the increased risk of poor outcomes for COPD patients following respiratory infection.

The emergence of severe acute respiratory syndrome coronavirus 2 (SARS-CoV-2) changed the epidemiology of respiratory infection and possibly the subsequent phenotype of AECOPD. Angiotensin-converting enzyme-2 receptors are upregulated in the airway and lung tissue of COPD patients,^10^ potentially making individuals more susceptible to SARS-CoV-2 virion uptake. Not only may infection risk increase in COPD patients, COPD is a significant risk factor for severe COVID-19 outcomes, such as hospitalisation, ICU stay and mortality. A meta-analysis of COVID-19 admissions reported that COPD carried an age-adjusted odds ratio (aOR) of 1.45 (95% confidence interval [95% CI]: 1.30–1.61) for hospitalisation, 1.28 (95% CI: 1.08–1.51) for ICU admission and 1.51 (95% CI: 1.37–1.65) for mortality^11^ compared with those without COPD. These earlier studies, however, had substantial limitations: COPD prevalence among COVID-19 patients varied from 0.0% to 32.9%. Patients were derived from the same hospitals and time periods, risking inadvertent inclusion of some patients more than once, which might distort prevalence and risk estimates. These studies, therefore, may not represent a cohort that is representative of the full patient population. Furthermore, most studies were retrospective and did not include non-COVID-19 comparison groups, making it difficult to interpret the full picture of AECOPD. Previously published studies also have not clearly defined if AECOPD due to SARS-CoV-2 infection has a worse prognosis than AECOPD due to other pathogens or with no infective component (non-infective exacerbation, NI-COPD), and whether any association varies by SARS-CoV-2 variant. Finally, the emergence of new SARS-CoV-2 variants, improved treatment and novel therapies for COVID-19, increasing physician experience and COVID-19 vaccine introduction may have changed the risk of severe outcomes following SARS-CoV-2 AECOPD.

To address these deficiencies in the literature, we analysed data collected during the first 21 months of an ongoing prospective cohort study to determine if hospitalisation with SARS-CoV-2 infection resulted in worse patient outcomes in COPD patients than hospitalisation with either non-SARS-CoV-2 infection AECOPD or NI-COPD. We sought to describe changes to the clinical phenotype of COPD exacerbation in hospitalised patients during different periods of the current COVID-19 pandemic, particularly during periods when different SARS-CoV-2 variants were predominant.

## Methods

### Study design

AvonCAP is a prospective observational cohort study undertaking comprehensive surveillance of adults admitted to two acute care hospitals encompassing all admissions in Bristol, UK (ISRCTN: 17354061). We analysed data from adult (≥18 y) hospitalised during 1 August 2020–4 May 2022, screening patients for signs and symptoms of community-acquired respiratory disease. Full study design details have been published previously.^12^ In this analysis, only patients with a confirmed respiratory exacerbation of COPD were included.^1^ AECOPD associated with hospital-acquired infection were excluded as aetiology and patient risk-factors may differ from community-acquired disease.

Demographic and clinical data were collected from patient records and recorded using REDCap.^13^ Data on co-morbidities at admission included Rockwood clinical frailty score (score of 5–9 indicating frailty)^14^ and Charlson Comorbidity Index (CCI).^15^ Vaccination records for each participant were obtained from linked general practitioner records. Admission severity scores, including CURB-65,^16^ Pneumonia Severity Index^17^ and NEWS-2,^18^ were also derived for each admission.

### Ethics and permission

The study was approved by the Health Research Authority Research Ethics Committee (East of England, Essex) (reference 20/EE/0157). Informed consent was obtained from cognisant patients, and declarations for participation were obtained from consultees for individuals lacking capacity. Patients who declined consent were not included in the analysis. If it was not practical to approach individuals for consent, data were included using approval from the Confidentiality Advisory Group under Section 251 of the 2006 NHS Act.

### Case definitions

Pre-existing COPD diagnosis at the time of admission was based on documented standard-of-care medical history. Aligning with NICE guidelines, AECOPD were defined as sustained symptom worsening from usual stable state (beyond normal day-to-day variations) with acute ( < 28 days) onset.^1^ Patients hospitalised with AECOPD and either a confirmed radiological/microbiological diagnosis of acute respiratory infection, in addition to those with respiratory infection diagnosed by the attending physician, were defined as having an infective AECOPD. Infective AECOPD were categorised into: (1) SARS-CoV-2 AECOPD, if there was a positive SARS-CoV-2 test on admission or <7 days prior to admission, using the UK Health Security Agency (UKHSA) diagnostic assay; or (2) non-SARS-CoV-2 infective AECOPD, if there was no positive SARS-CoV-2 test.^12^ Following medical chart review, AECOPD patients with no positive SARS-CoV-2 test and no radiological, microbiological or clinical diagnosis of respiratory infection were classified as NI-COPD.

### Study objectives

The primary objective was to determine if hospitalisation with AECOPD due to SARS-CoV-2 infection resulted in worse patient outcomes compared with hospitalisation due to non-SARS-CoV-2 infective AECOPD or NI-COPD. Secondary objectives included describing the clinical phenotype of AECOPD attributable to SARS-CoV-2, non-SARS-CoV-2 infection and NI-COPD over time following variant of concern (VOC) emergence.

### Statistical analysis

Comparisons between SARS-CoV-2 AECOPD, non-SARS-CoV-2 infective AECOPD and NI-COPD were made using Kruskal-Wallis or Pearson’s chi-square tests, as appropriate to the nature and distribution of each variable. Categorical data are presented as counts and percentages, and continuous data as medians with interquartile ranges (IQRs).

We used Cox proportional regression models for analysis of both 30-day mortality and length of hospital stay (LOS), right-censoring patients who, respectively, survived and died within 30 days of admission. Cox proportional regression analysis of LOS was used, as this estimated the increase in hazard ratio of a patient not being discharged and, thus, having a longer stay in hospital. For analysis of positive pressure support, we used a logistic regression model. In all models, the reference group was non-SARS-CoV-2 infective AECOPD. We included the following confounders in all three models: age, sex, CCI class, current smoking status, eosinopenia, consolidation, acidaemia and atrial fibrillation (ECAF) score (dyspnoea, eosinopenia, consolidation, acidaemia and atrial fibrillation [DECAF] score without extended Medical Research Council dyspnoea score [eMRCD] as this was unavailable),^19^ and whether the patient was immunodeficient or using inhaled corticosteroids. These confounders were decided *a priori*. Either adjusted hazard ratio (aHR) or aOR with 95% CI was determined for each variable. We performed a sensitivity analysis, restricting analyses to time-periods when specific VOCs dominated (Supplementary Data 1), thereby adjusting for time-dependent variables including healthcare resource usage and demand, public health interventions, available COVID-19 therapies, COVID-19 vaccination programme stage and severity of the dominant variant. We also carried out subgroup analyses by age stratification (with groups being aged over and under 65 year olds) and sex. Finally, we also performed propensity score matching as a sensitivity analysis; logistic regression on all covariates is used to define propensity scores, followed by matching using optimal pair matching (Supplementary Data 2). Missing data points were omitted from the analyses. In all analyses, *p* < 0.05 was considered statistically significant. Statistical analyses were conducted using R version 4.1.0.^20^

## Results

**Figure 1.**
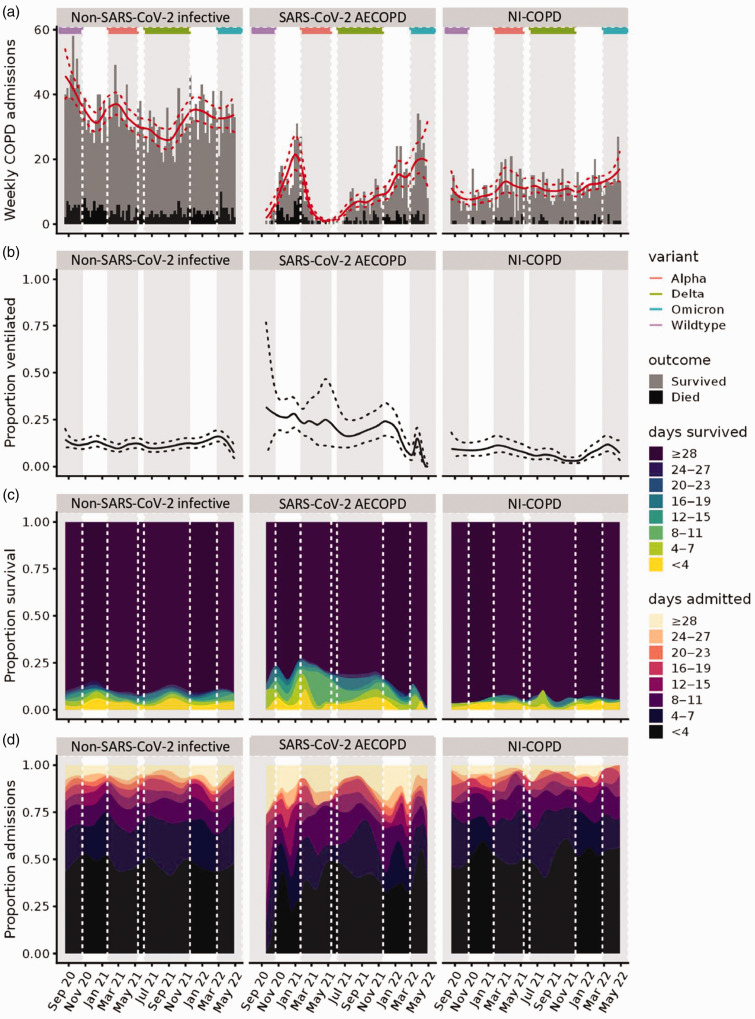
Admissions, positive pressure support, 30-day mortality and length of hospital stay over time, shown by three COPD groups (infective, SARS-CoV-2, non-infective).COPD: chronic obstructive pulmonary disease; SARS-CoV-2: severe acute respiratory syndrome coronavirus 2; AECOPD: acute exacerbations of chronic obstructive pulmonary disease.

During the study period, 19,802 admissions with acute lower respiratory tract disease occurred, including 4848 with AECOPD: 816 SARS-CoV-2 AECOPD, 3038 non-SARS-CoV-2 infective AECOPD and 994 NI-COPD hospitalisations (Supplementary Data 3). Patients hospitalised with AECOPD were elderly (median [IQR] age 75 y [67–82]); frequently had co-morbidities (64% had a CCI score >4) and were frail (63% had Rockwood score ≥5). Positive pressure support was more frequently required in SARS-CoV-2 AECOPD compared with non-SARS-CoV-2 infective AECOPD and NI-COPD (18.5% and 11.7% vs. 7.5%, respectively). Despite similar age, CCI and frailty scores, patients admitted with SARS-CoV-2 AECOPD had longer hospitalisation stays (median 7 [3–15] days vs. 5 [2–10] and 4 [2–9]) and increased 30-day mortality (16.9% vs. 11.1% and 5.9%) than those with non-SARS-CoV-2 infective AECOPD or NI-COPD, respectively (Table 1, Figure 1, Supplementary Data 4).

**Table 1.** Characteristics and outcomes of adults hospitalised with AECOPD.

Characteristic	Overall	SARS-CoV-2 AECOPD	Non-SARS-CoV-2 infective AECOPD	NICOPD	
*n* (%)	*n* = 4848	*n* = 816	*n* = 3038	*n* = 994	*p* value^a^
*Demographics*					
Age at admission (years)					0.048
Median (IQR)	75 (67, 82)	75 (68, 83)	75 (66, 82)	74 (67, 82)	
Sex					0.8
Male	2471 (51.0%)	421 (51.6%)	1552 (51.1%)	498 (50.1%)	
Female	2377 (49.0%)	395 (48.4%)	1486 (48.9%)	496 (49.9%)	
Ethnicity					<0.001
White British	4079 (84.1%)	669 (82.0%)	2591 (85.3%)	819 (82.4%)	
White other	104 (2.1%)	19 (2.3%)	58 (1.9%)	27 (2.7%)	
Unknown	541 (11.2%)	94 (11.5%)	326 (10.7%)	121 (12.2%)	
Care home resident					0.061
No	3759 (77.5%)	627 (76.8%)	2364 (77.9%)	768 (77.3%)	
Yes	344 (7.1%)	60 (7.4%)	232 (7.6%)	52 (5.2%)	
Unknown	745 (15.5%)	129 (16.2%)	442 (14.5%)	174 (17.7%)	
Smoking status					<0.001
Non-smoker	575 (11.9%)	105 (12.9%)	352 (11.6%)	118 (11.9%)	
Current smoker	991 (20.4%)	114 (14.0%)	665 (21.9%)	212 (21.3%)	
Ex-smoker	3282 (67.7%)	597 (73.2%)	2,021 (66.5%)	664 (66.8%)	
COVID-19 vaccine doses					<0.001
Unvaccinated	1793 (37.0%)	337 (41.2%)	1150 (37.9%)	306 (30.8%)	
1 dose	569 (11.7%)	67 (8.2%)	375 (12.3%)	127 (12.8%)	
2 doses	1416 (29.2%)	183 (22.4%)	911 (30.0%)	322 (32.5%)	
3 doses	972 (20.1%)	206 (25.2%)	547 (18.0%)	219 (22.1%)	
4 doses	96 (2.0%)	23 (2.8%)	55 (1.8%)	18 (1.8%)	
Unknown	2 (0.0%)	0 (0%)	0 (0%)	2 (0.2%)	
*Clinical scores*					
CCI score					0.03
Not severe ( < 5)	1756 (36.2%)	264 (32.4%)	1135 (37.4%)	357 (35.9%)	
Severe (≥5)	3092 (63.8%)	552 (67.6%)	1903 (62.6%)	637 (64.1%)	
Rockwood frailty score					0.4
Rockwood <5	1771 (36.5%)	282 (34.6%)	1120 (36.9%)	369 (37.1%)	
Rockwood ≥5	3077 (63.5%)	534 (65.4%)	1918 (63.1%)	625 (62.9%)	
CURB-65 score					0.1
0 (Low risk)	735 (15.2%)	105 (12.9%)	483 (15.9%)	147 (14.8%)	
1 (Low risk)	2630 (54.2%)	443 (54.3%)	1613 (53.1%)	574 (57.7%)	
2 (Intermediate risk)	1249 (25.8%)	229 (28.1%)	786 (25.9%)	234 (23.5%)	
3 (High risk)	212 (4.4%)	36 (4.4%)	140 (4.6%)	36 (3.6%)	
4 (High risk)	22 (0.5%)	3 (0.4%)	16 (0.5%)	3 (0.3%)	
*Co-morbidities*					
IHD					0.006
No	3978 (82.1%)	638 (78.2%)	2520 (82.9%)	820 (82.5%)	
Yes	870 (17.9%)	178 (21.8%)	518 (17.1%)	174 (17.5%)	
Hypertension^b^					0.4
No	4055 (83.6%)	670 (82.1%)	2550 (83.9%)	835 (84.0%)	
Yes	793 (16.4%)	146 (17.9%)	488 (16.1%)	159 (16.0%)	
Congestive cardiac failure					<0.001
No	3968 (81.8%)	668 (81.9%)	2543 (83.7%)	757 (76.2%)	
Yes	880 (18.2%)	148 (18.1%)	495 (16.3%)	237 (23.8%)	
Immunodeficiency					<0.001
No	4006 (82.6%)	712 (87.3%)	2489 (81.9%)	805 (81.0%)	
Yes	842 (17.4%)	104 (12.7%)	549 (18.1%)	189 (19.0%)	
Diabetes mellitus					0.3
None	3751 (77.4%)	619 (75.9%)	2361 (77.7%)	771 (77.6%)	
Type 1	40 (0.8%)	6 (0.7%)	30 (1.0%)	4 (0.4%)	
Type 2	1057 (21.8%)	191 (23.4%)	647 (21.3%)	219 (22.0%)	
CKD^c^					0.057
No	3647 (75.3%)	602 (73.8%)	2315 (76.2%)	730 (73.6%)	
Mild	1008 (20.8%)	171 (21.0%)	608 (20.0%)	229 (23.1%)	
Moderate/severe	191 (3.9%)	43 (5.3%)	115 (3.8%)	33 (3.3%)	
Taking inhaled corticosteroids					0.6
No	1984 (40.9%)	334 (41.0%)	1229 (40.4%)	421 (41.9%)	
Yes	2856 (58.9%)	480 (58.9%)	1805 (59.5%)	571 (57.9%)	
Unknown	8 (0.2%)	2 (0.2%)	4 (0.1%)	2 (0.2%)	
*Outcomes*					
30-day mortality					<0.001
Survived	4314 (89.0%)	678 (83.1%)	2701 (88.9%)	935 (94.1%)	
Died	534 (11.0%)	138 (16.9%)	337 (11.1%)	59 (5.9%)	
Length of hospital stay (days)	5 (2, 11)	7 (3, 15)	5 (2, 10)	4 (2, 9)	<0.001
Positive pressure support					<0.001
No	4267 (88.0%)	665 (81.5%)	2683 (88.3%)	919 (92.5%)	
Yes	581 (12.0%)	151 (18.5%)	355 (11.7%)	75 (7.5%)	

AECOPD: acute exacerbations of chronic obstructive pulmonary disease; PPV23: 23-valent pneumococcal polysaccharide vaccine; PCV13: 13-valent pneumococcal conjugate vaccine; IHD: ischaemic heart disease; CCI: Charlson Comorbidity Index; CKD: chronic kidney disease; CVA: cerebrovascular accident; *n*: number; NI-COPD: non-infective exacerbation of COPD; TIA: transient ischaemic attack; IQR: interquartile range.

a Kruskal-Wallis rank sum test; Pearson's chi-square test.

b Hypertension was only included if causing other cardiac complications.

c Chronic kidney disease (CKD) was classified as mild if stage 1–3; moderate/severe if stage 4–5, end-stage renal failure or there was dialysis dependence.

**Figure 2.**
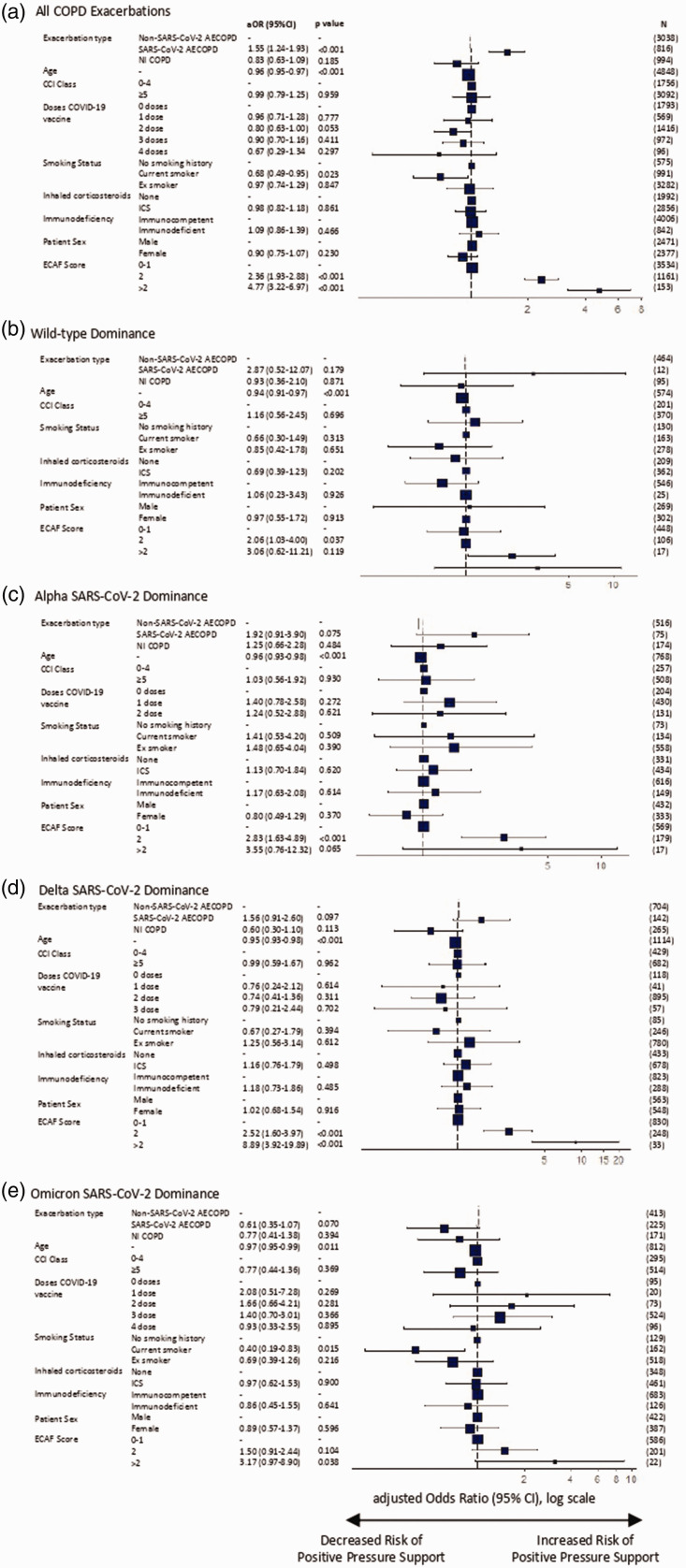
Logistic regression analysis of requirement for positive pressure ventilatory support in patients hospitalised with COPD exacerbations. Plots of adjusted odds ratio for (a) all COPD admissions, and admissions during time-periods of (b) wild-type, (c) Alpha, (d) Delta and (e) Omicron variant domination. Results are presented as aOR (95% CI) on a log scale, with *p* value provided for each value (Supplementary Table 4).COPD: chronic obstructive pulmonary disease; SARS-CoV-2: severe acute respiratory syndrome coronavirus 2; CCI: Charlson Comorbidity Index; AECOPD, acute exacerbations of chronic obstructive pulmonary disease; CI: confidence interval; ICS: inhaled corticosteroids; N: number; NI-COPD: non-infectious COPD exacerbation; OR: odds ratio.

**Figure 3. fig3-01410768231184162:**
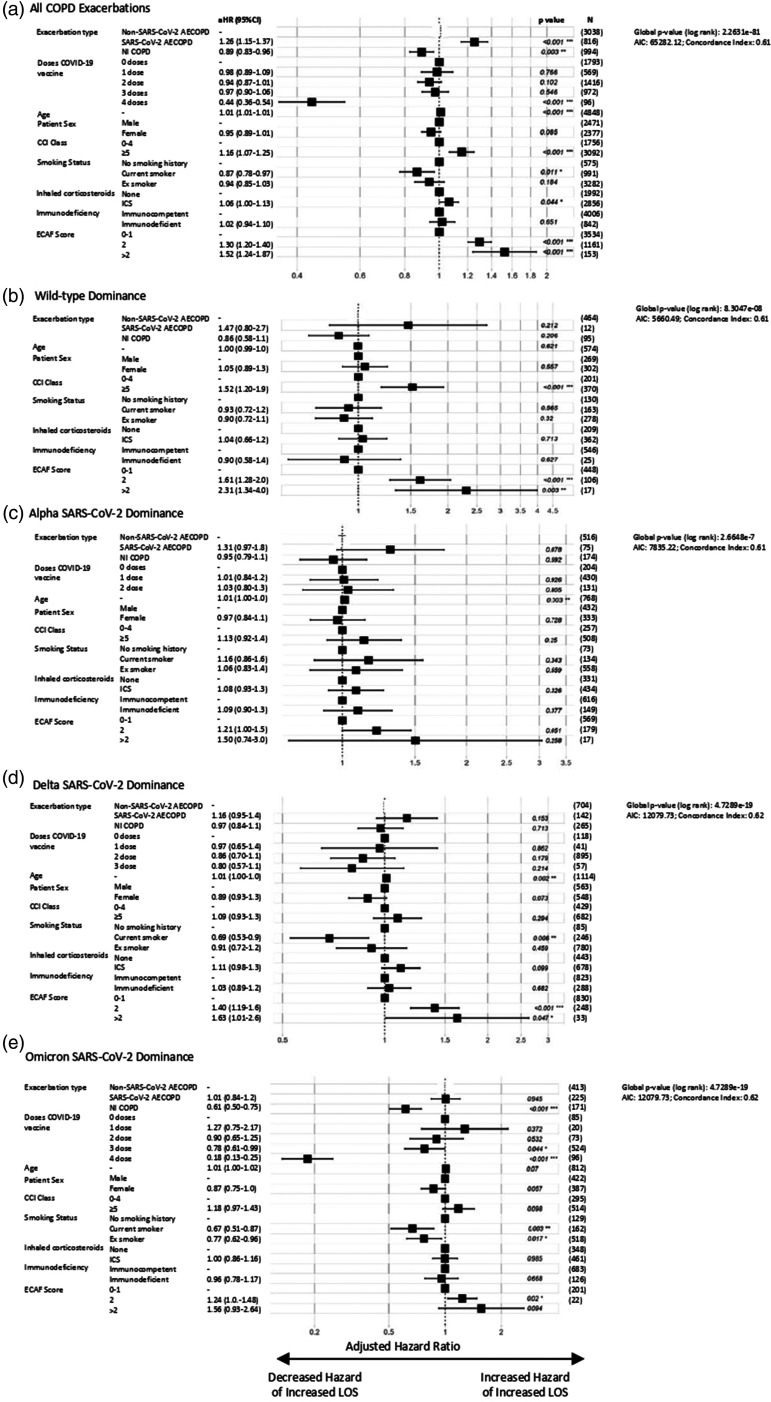
Cox regression analysis of hospital length of stay in patients hospitalised with COPD exacerbations. Plots of aHR for (a) all COPD admissions, and admissions during time-periods of (b) wild-type, (c) Alpha, (d) Delta and (e) Omicron variant domination. Results are presented as aHR (95% CI) on a log scale, with *p* value provided for each value, in addition to global *p* value (log rank), Akaike information criterion (AIC) and Concordance Index (Supplementary Table 5).AECOPD: acute exacerbations of chronic obstructive pulmonary disease; CCI: Charlson Comorbidity Index; CI: confidence interval; aHR: adjusted hazard ratio; ICS: inhaled corticosteroids; N: number; NI-COPD: non-infectious COPD exacerbation.

In adjusted regression models, relative to non-SARS-CoV-2 infective AECOPD, patients hospitalised with SARS-CoV-2 AECOPD were more likely to require positive pressure support (aOR = 1.55 [95% CI: 1.24–1.93]), with increased hazard of both longer hospital LOS (aHR = 1.26 [95% CI: 1.15–1.37]) and 30-day mortality (aHR = 1.35 [95% CI: 1.10–1.65]). Patients hospitalised with NI-COPD had a reduced hazard of a longer hospital LOS (aHR = 0.89 [95% CI: 0.83–0.96]) and 30-day mortality (aHR = 0.75 [95% CI: 0.56–1.00]) than non-SARS-CoV-2 infective AECOPD, with a non-significant reduction in risk of positive pressure support (aOR = 0.83 [95% CI: 0.63–1.09]) (Figures 2 [Fig fig3-01410768231184162]to 4, Supplementary Data 5–7). The hazard of prolonged hospital admission and 30-day mortality in hospitalised AECOPD patients was reduced in individuals who had received SARS-CoV-2 vaccination: successive doses having greater risk reductions. COPD patients taking an inhaled corticosteroid had increased risk of 30-day mortality compared with those who were not (associated aHR = 1.56 [95% CI: 1.30–1.87]); however, there was no change in risk for either positive pressure support or longer hospitalisation in patients using an inhaled corticosteroid (Supplementary Data 5–7). This may be explained by COPD severity and phenotype differences in these patients (Supplementary Data 8). Propensity scores before and after optimal matching are shown in Supplementary Data 2; trends in clinical outcomes remained after matching (Supplementary Data 9). From the subgroup analyses stratified by age and sex, we found that the effect of COVID-induced exacerbations on all three clinical outcomes was statistically significant for males, but not for females (apart from length of hospitalisation), as well as for patients over 65 years of age, but not under 65 (Supplementary Data 10–15).

**Figure 4.**
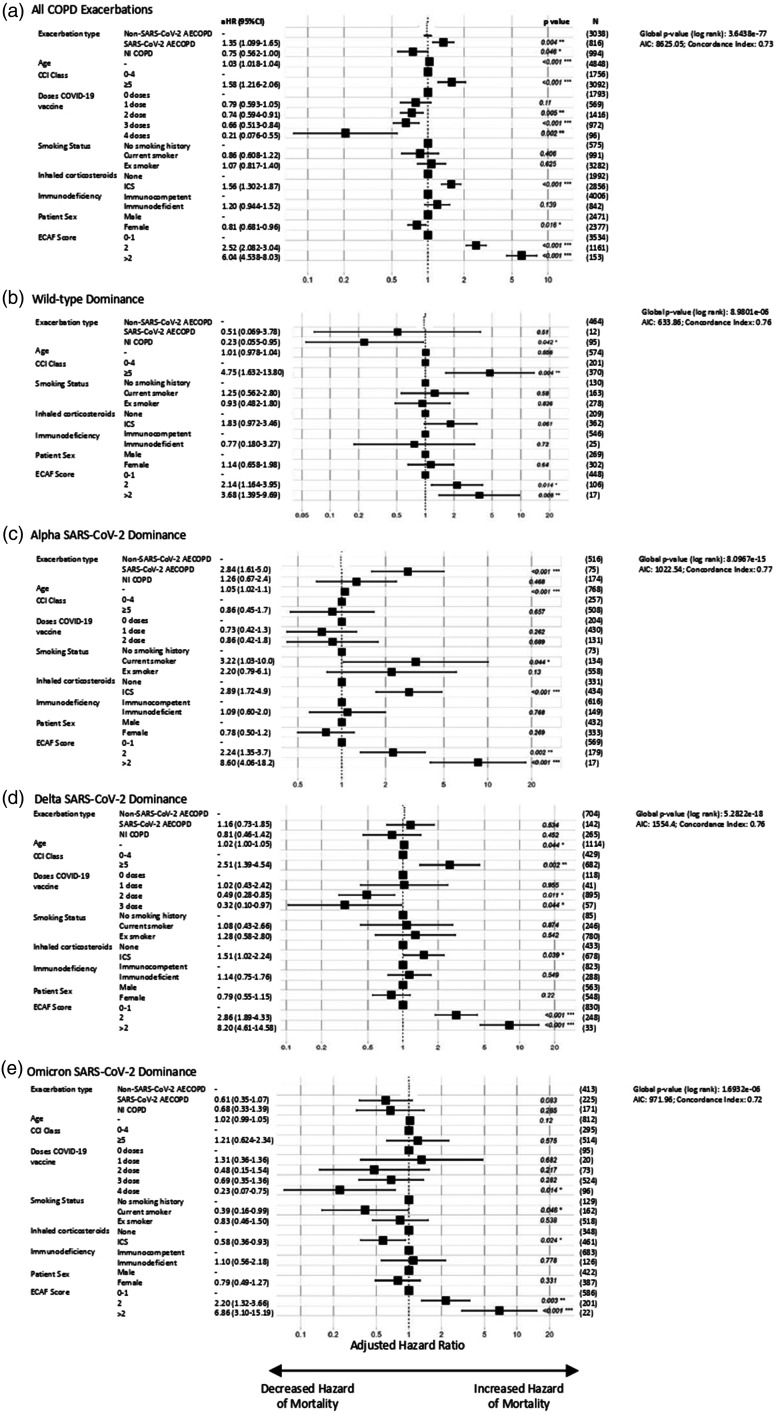
Cox regression analysis of 30-day mortality in patients hospitalised with COPD exacerbations. Plots of aHR for (a) all COPD admissions, and admissions during time-periods of (b) wild-type, (c) Alpha, (d) Delta and (e) Omicron variant domination. Results are presented as aHR (95% CI) on a log scale, with *p* value provided for each value, in addition to global *p* value (log rank), Akaike information criterion (AIC) and Concordance Index (Supplementary Table 6).AECOPD: acute exacerbations of chronic obstructive pulmonary disease; CCI: Charlson Comorbidity Index; CI: confidence interval; aHR: adjusted hazard ratio; ICS: inhaled corticosteroids; N: number; NI-COPD: non-infectious COPD exacerbation.

In adjusted regression models restricted to time-periods when specific VOCs were predominant (Figures 2(b) to (e), 3(b) to (e) and 4(b) to (e), Supplementary Data 16), individuals with SARS-CoV-2 AECOPD saw improved outcomes over time, relative to those hospitalised with non-SARS-CoV-2 AECOPD. During the Alpha dominance, all three measured outcomes showed a trend towards a poorer prognosis for SARS-CoV-2 AECOPD than those with exacerbations caused by other infectious agents, with statistical significance only demonstrated for increased 30-day mortality risk in SARS-CoV-2 AECOPD versus non-SARS-CoV-2 infection AECOPD. When Omicron dominated circulation, SARS-CoV-2 AECOPD appeared to result in better clinical outcomes than non-SARS-CoV-2 infectious AECOPD, although this trend was not statistically significant.

## Discussion

COPD is recognised as a significant risk factor for hospitalisation, ICU stay and mortality with COVID-19.^11^ To our knowledge, this is the first prospective study comparing the risk of poor outcomes in patients hospitalised with SARS-CoV-2 infective AECOPD, non-SARS-CoV-2 infective AECOPD and NI-COPD. We found that SARS-CoV-2 AECOPD were associated with worse outcomes than for those admitted with non-SARS-CoV2 infective AECOPD, with a 55%, 26% and 35% increased risk of positive pressure support, hospitalisation length and 30-day mortality, respectively, when controlling for potential confounders such as age, chronic medical conditions and increased vaccination coverage over time. We found that patient outcomes associated with SARS-CoV-2 AECOPD changed over time and specific VOCs, with improved outcomes when the Omicron variant was circulating. Overall, 11% hospitalised with SARS-CoV-2 AECOPD died within 30 days of admission. These results show that SARS-CoV-2 emergence has resulted in increased risk of clinically relevant adverse outcomes in hospitalised COPD patients, including positive pressure support requirements, with important resource implications for healthcare systems. Nevertheless, 83% AECOPD hospitalisations found in this study did not involve SARS-CoV-2 and were associated with a 30-day mortality of 9.8%, highlighting the pervasive burden of non-SARS-CoV-2 AECOPD admissions throughout the pandemic.

This study has many strengths and limitations, as discussed previously.^12,21,22^ By undertaking a prospective, comprehensive case ascertainment and assessment, and gathering complete data, including detailed data on AECOPD phenotype and linked community records, we obtain a comparatively rich, novel and accurate dataset. Our study design ensures full case ascertainment, thereby reducing bias by minimising exclusion of vulnerable and hard-to-reach patient groups. UK healthcare, including vaccination programmes, is provided at no cost at the point of delivery; hence, our cohort is not biased by issues that are seen in fee-based systems. This study spans multiple COVID-19 waves representing periods of different variant dominance, and each VOC varies in ability to transmit and cause severe disease.^23–25^ Conducting a sensitivity analysis restricting participants to specific time periods allows us to provide some assurance that we have adjusted for factors that may vary over time but are typically difficult to control in real-world studies, including patient treatment preferences, novel COVID-19 therapies and healthcare pressure. Over the 21 months analysed, we only observe patient outcomes after hospitalisation. AECOPD severity was undoubtedly affected by the emergence of a new pathogen, public health interventions and other factors that are difficult to quantify. While the Bristol population is representative of the UK population, it is principally Caucasian, and our findings must be interpreted in this context. Previous SARS-CoV-2 exposure could not be determined, and this may have impacted on our findings, although the magnitude of any such effect is unclear. We could not determine the baseline respiratory function of COPD patients in this cohort, partly due to reduced spirometry testing during the pandemic. We therefore could not determine and adjust by COPD severity scores including Global Initiative for Chronic Obstructive Lung Disease criteria, BODE Index or complete DECAF score (as eMRCD was unavailable). We did not collect data on medication prescription or adherence, with the exception of inhaled corticosteroids, and are unable to adjust by use of medication such as beta-blockers or angiotensin-converting enzyme inhibitor. We also did not collect data on treatment escalation thresholds, and these may have affected this analysis. Additionally, to prioritise SARS-CoV-2 testing, study hospitals undertook limited microbiological testing for other respiratory pathogens and, using standard-of-care results, we are unable to comment on the microbial aetiology of non-SARS-CoV-2 respiratory infection and we acknowledge that different pathogens may be associated with different severities.

Our findings align with other analyses highlighting the increased risk of severe COVID-19 and poor outcomes in COPD patients; however, patients in this cohort had a higher mortality (11.0%) than the 3.6% found in a pre-pandemic national audit.^26^ A previous meta-analysis found that the heterogeneity in COVID-19 effects on COPD patients on hospitalisation, ICU admission and mortality was not fully explained by differences in age or sex distribution of patients.^27^ A second, larger and more recent meta-analysis found that COPD patients were at higher risk of ICU admission (aOR = 1.28, 95% CI: 1.08–1.51), intubation (aOR = 1.45, 95% CI: 1.30–1.61) and mortality (aOR = 1.41, 95% CI: 1.37–1.65) than those without COPD.^11^ Both meta-analyses found substantial heterogeneity between studies that could not be fully adjusted for within available data. Reassuringly, our estimate of 30-day mortality risk associated with SARS-CoV-2 AECOPD is comparable to that in the meta-analyses. However, the estimated increase in risk of positive pressure support associated with SARS-CoV-2 AECOPD in our analysis is slightly higher than that for intubation in the meta-analyses. The risk of positive pressure support will likely be higher than invasive intubation because it is a less invasive procedure, which is more commonly used, and therefore this finding validates our results.^28–30^ These findings should provide useful insight into the resources required to continue to provide appropriate medical treatment in secondary care settings. However, measures implemented during the period analysed here aimed at reducing respiratory pathogen transmission,^28^ increasing medication adherence^29^ and smoking cessation^30^ may have affected the disease phenotype profile of this cohort and this may affect healthcare resource calculations. Given that a 50% (range 27%–78%) reduction in AECOPD hospitalisations during the study period compared with pre-pandemic time periods has been reported,^28^ this may need to be factored into estimating future healthcare resource requirements.

We found evidence suggesting that outcomes of patients hospitalised with SARS-CoV-2 AECOPD improved over time, as circulating and dominant variants, vaccination rates, healthcare pressure and COVID-19 treatments varied. Overall, our data show that Alpha infection had the greatest impact on positive pressure support, LOS and mortality within this cohort. By Omicron dominance, SARS-CoV-2 AECOPD appeared to result in better clinical outcomes for patients hospitalised with COPD exacerbation, highlighting a trend towards lower risk of severe disease in hospitalised COPD patients as successive variants emerged and the pandemic progressed.^23^ Previous studies suggest that Omicron may be associated with less severe infection than Delta.^21,23,24^ Successful vaccination programmes and changes in healthcare including new COVID-19 treatments may account for some of the reduced severity of Omicron seen here. Contrasting with our results, a systematic review of the severity of infection in all patients with Alpha, Beta, Gamma or Delta SARS-CoV-2 concluded that the Alpha was associated with a higher risk of severe disease than wild-type virus, and Delta cases had a significantly higher risk of hospitalisation, ICU admission and death compared with wild-type and Alpha.^25^

COVID-19 vaccination was associated with reduced risk of prolonged hospital admission, positive pressure support and mortality within 30 days of hospitalisation in COPD patients overall and in all periods of different variant dominance, though strong evidence was only found for 30-day mortality, with successive doses showing greater risk reductions. These results highlight that COVID-19 vaccination has an important effect in modifying risk of poor health outcome in COPD patients. Previous analyses of SARS-CoV-2 disease severity have shown that COVID-19 vaccination is independently associated with lower in-hospital disease severity.^21,23,24^ Since COVID-19 vaccination status modifies severe disease risk, we used this variable to adjust for the risk of adverse clinical outcome in this analysis. However, we did not seek to provide an estimate of vaccine effectiveness against these outcomes and the analysis presented here does not attempt to do this. In line with the UK COVID-19 vaccination programme, individuals in this cohort will have become eligible for vaccination at different periods of time and access to vaccination may have changed. Therefore, the population receiving vaccination over time is not static and changed throughout the course of this 21-month study. Further, an individual’s vaccination status may be confounded by other factors that we have not adjusted for, such as access to vaccination or other healthcare facilities. By the time of Omicron dominance, the vaccination programme was established, and individuals admitted who have received fewer than three doses (including some unvaccinated) may differ from those who have received more than two COVID-19 vaccines at admission (Supplementary Data 16). Therefore, the results presented in this analysis should not be interpreted as estimates of vaccine effectiveness in patients hospitalised with AECOPD.

The increased need for positive pressure support, longer hospital admissions and worse 30-day mortality in SARS-CoV-2-related AECOPD patients found in this analysis indicates that SARS-CoV-2 AECOPD has worse patient outcomes following hospitalisation than both non-SARS-CoV-2 AECOPD and NI-COPD. Characterising the nature of a COPD exacerbation therefore appears to provide important information about which patients are at risk of worse outcomes. As the pandemic progressed, there was a trend towards a reducing risk of severe outcome from SARS-CoV-2 AECOPD compared with non-SARS-CoV-2 AECOPD and NI-COPD. During Omicron variant dominance, this analysis suggests that SARS-CoV-2 AECOPD may be less severe than other infectious AECOPD. Nevertheless, the majority of AECOPD admissions were not related to SARS-CoV-2, and there was a 9.8% 30-day mortality rate among non-SARS-CoV-2 COPD patients. While progress through the pandemic has improved outcomes for COPD patients, our analysis suggests that COPD exacerbation-related morbidity and mortality remain substantial. Overall, these data highlight the significant resources required to appropriately manage COPD patients and should contribute to public health and healthcare resource planning.

## Supplemental Material

sj-pdf-1-jrs-10.1177_01410768231184162 - Supplemental material for Impact of SARS-CoV-2 infective exacerbation of chronic obstructive pulmonary disease on clinical outcomes in a prospective cohort study of hospitalised adults Click here for additional data file. Supplemental material, sj-pdf-1-jrs-10.1177_01410768231184162 for Impact of SARS-CoV-2 infective exacerbation of chronic obstructive pulmonary disease on clinical outcomes in a prospective cohort study of hospitalised adults by Catherine Hyams, George Qian, George Nava, Robert Challen, Elizabeth Begier, Jo Southern, Maria Lahuerta, Jennifer L Nguyen, Jade King, Anna Morley, Madeleine Clout, Nick Maskell, Luis Jodar, Jennifer Oliver, Gillian Ellsbury, John M McLaughlin, Bradford D Gessner, Adam Finn, Leon Danon, James W Dodd and The Avon CAP Research Group in Journal of the Royal Society of Medicine

sj-pdf-2-jrs-10.1177_01410768231184162 - Supplemental material for Impact of SARS-CoV-2 infective exacerbation of chronic obstructive pulmonary disease on clinical outcomes in a prospective cohort study of hospitalised adults Click here for additional data file. Supplemental material, sj-pdf-2-jrs-10.1177_01410768231184162 for Impact of SARS-CoV-2 infective exacerbation of chronic obstructive pulmonary disease on clinical outcomes in a prospective cohort study of hospitalised adults by Catherine Hyams, George Qian, George Nava, Robert Challen, Elizabeth Begier, Jo Southern, Maria Lahuerta, Jennifer L Nguyen, Jade King, Anna Morley, Madeleine Clout, Nick Maskell, Luis Jodar, Jennifer Oliver, Gillian Ellsbury, John M McLaughlin, Bradford D Gessner, Adam Finn, Leon Danon, James W Dodd and The Avon CAP Research Group in Journal of the Royal Society of Medicine

sj-xlsx-3-jrs-10.1177_01410768231184162 - Supplemental material for Impact of SARS-CoV-2 infective exacerbation of chronic obstructive pulmonary disease on clinical outcomes in a prospective cohort study of hospitalised adults Click here for additional data file. Supplemental material, sj-xlsx-3-jrs-10.1177_01410768231184162 for Impact of SARS-CoV-2 infective exacerbation of chronic obstructive pulmonary disease on clinical outcomes in a prospective cohort study of hospitalised adults by Catherine Hyams, George Qian, George Nava, Robert Challen, Elizabeth Begier, Jo Southern, Maria Lahuerta, Jennifer L Nguyen, Jade King, Anna Morley, Madeleine Clout, Nick Maskell, Luis Jodar, Jennifer Oliver, Gillian Ellsbury, John M McLaughlin, Bradford D Gessner, Adam Finn, Leon Danon, James W Dodd and The Avon CAP Research Group in Journal of the Royal Society of Medicine

sj-pdf-4-jrs-10.1177_01410768231184162 - Supplemental material for Impact of SARS-CoV-2 infective exacerbation of chronic obstructive pulmonary disease on clinical outcomes in a prospective cohort study of hospitalised adults Click here for additional data file. Supplemental material, sj-pdf-4-jrs-10.1177_01410768231184162 for Impact of SARS-CoV-2 infective exacerbation of chronic obstructive pulmonary disease on clinical outcomes in a prospective cohort study of hospitalised adults by Catherine Hyams, George Qian, George Nava, Robert Challen, Elizabeth Begier, Jo Southern, Maria Lahuerta, Jennifer L Nguyen, Jade King, Anna Morley, Madeleine Clout, Nick Maskell, Luis Jodar, Jennifer Oliver, Gillian Ellsbury, John M McLaughlin, Bradford D Gessner, Adam Finn, Leon Danon, James W Dodd and The Avon CAP Research Group in Journal of the Royal Society of Medicine

## Declarations

### Guarantor

CH.

### Data sharing

The data used in this study are sensitive and cannot be made publicly available without breaching patient confidentiality rules. Therefore, individual participant data and a data dictionary are not available to other researchers.

### The AvonCAP Research Group

Amelia Langdon, Anabella Turner, Anya Mattocks, Bethany Osborne, Charli Grimes, Claire Mitchell, David Adegbite, Emma Bridgeman, Emma Scott, Fiona Perkins, Francesca Bayley, Gabriella Ruffino, Gabriella Valentine, Grace Tilzey, Jane Kinney, Johanna Kellett Wright, Julia Brzezinska, Julie Cloake, Katarina Milutinovic, Kate Helliker, Katie Maughan, Kazminder Fox, Konstantina Minou, Lana Ward, Leah Fleming, Leigh Morrison, Lily Smart, Louise Wright, Lucy Grimwood, Maddalena Bellavia, Madeleine Clout, Marianne Vasquez, Milo Jeenes-Flanagan, Natalie Chang, Niall Grace, Maria Garcia Gonzalez, Nicola Manning, Oliver Griffiths, Pip Croxford, Peter Sequenza, Rajeka Lazarus, Rhian Walters, Robin Marlow, Robyn Heath, Rupert Antico, Sandi Nammuni Arachchge, Seevakumar Suppiah, Taslima Mona, Tawassal Riaz, Vicki Mackay, Zandile Maseko, Zoe Taylor, Zsuzsa Szasz-Benczur, Zsolt Friedrich.

### Provenance

Not commissioned; peer reviewed by Jeremy Man Ho Hui and Julie Morris.
