# Supplementary material for: Impact of SARS-CoV-2 infective exacerbation of chronic obstructive pulmonary disease on clinical outcomes in a prospective cohort study of hospitalised adults
Source: J R Soc Med. 2023 Jul 5;116(11):371–85. doi: 10.1177/01410768231184162 (PMC10686205; doi:10.1177/01410768231184162)

Supplementary Data 1: Observed counts of lineages of SARS-CoV-2 in Bristol UK

(A) Proportion of cases of each lineage as sequenced by the Lighthouse Labs, covering most of Pillar 2 testing.

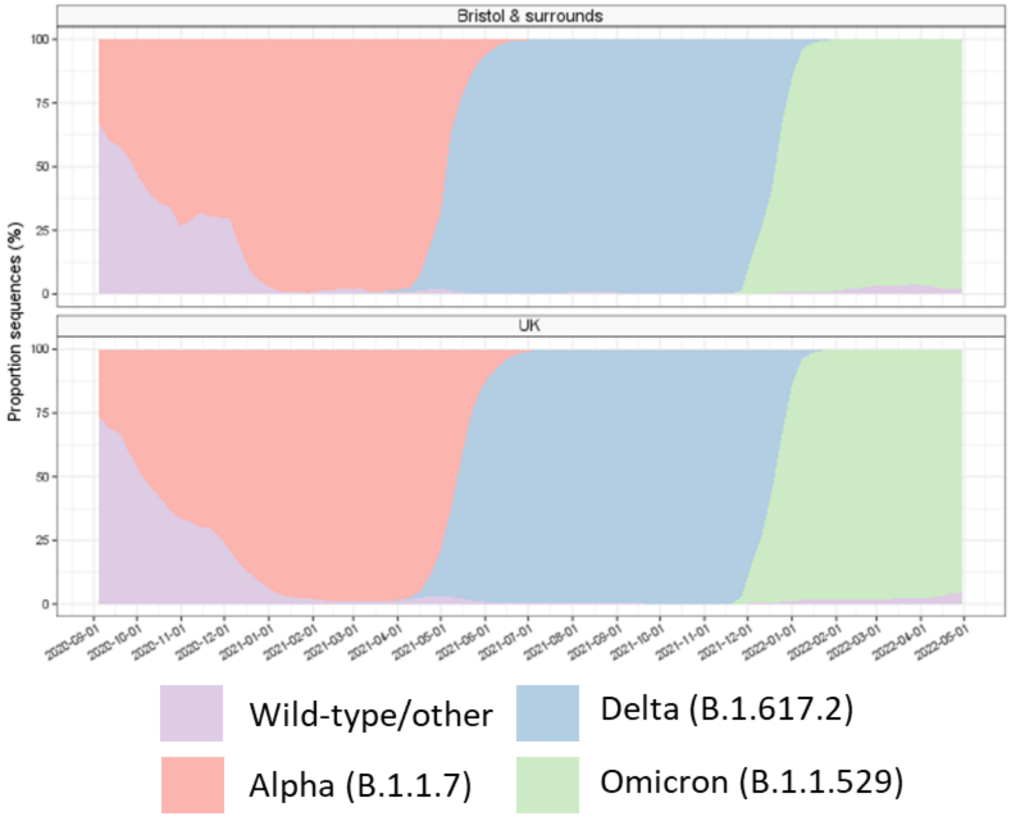

(B) Dates used in the study to restrict analysis to periods of time when each variant was dominant in circulation.

Variant or Study Window	Start Date	End date
Whole Study	1st August 2020	4th May 2022
Wild-type	1st August 2020	17th October 2020
Alpha (B.1.1.7)	17th January 2021	1st May 2021
Delta (B.1.617.2)	1st June 2021	7th November 2021
Omicron (B.1.1.529 and B.1.1.529 BA.2)	7th February 2022	4th May 2022

Supplementary Data 2: Propensity score distribution before and after optimal matching.

Distribution of propensity scores (A) before and (B) after optimal propensity score matching. Smaller circles indicate 1:1 matching between cases and controls. Circle sizes indicate the assigned weights for group comparison.

A

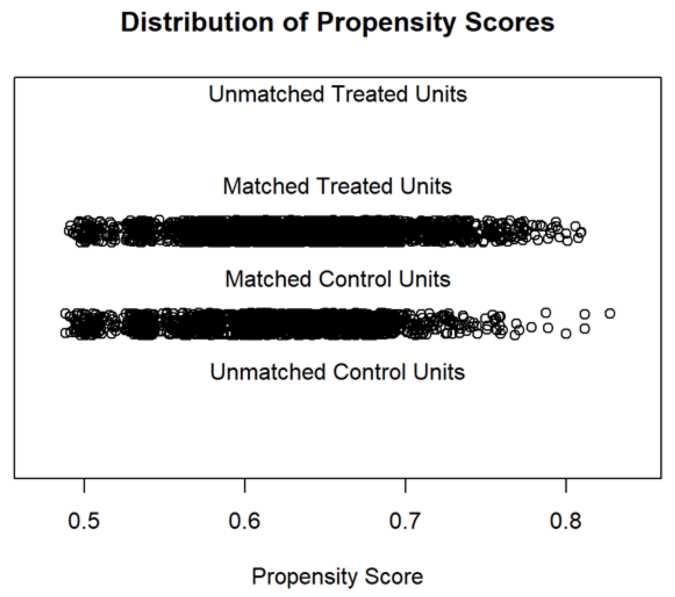

B

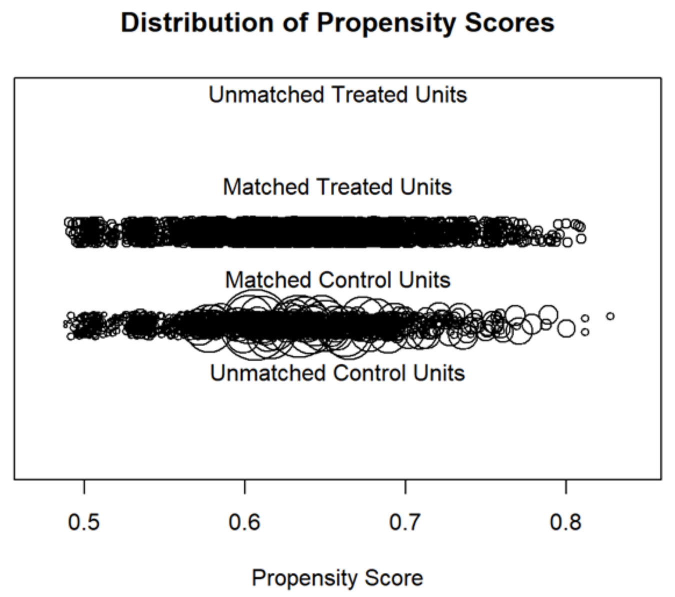

Supplementary Data 3: Study Flow Diagram

Of the 182,677 episodes of adults hospitalised during the study period, 4,848 adults were admitted with an acute exacerbation of COPD. This represents 817 admissions with SARS-CoV-2 AECOPD, 3,041 with non-SARS-CoV-2 infective AECOPD and 997 admissions with 997 NI-COPD

aLRTD, acute lower respiratory tract disease; AECOPD, acute exacerbation of COPD; NI-COPD, non-infective COPD exacerbation; PE, pulmonary embolus

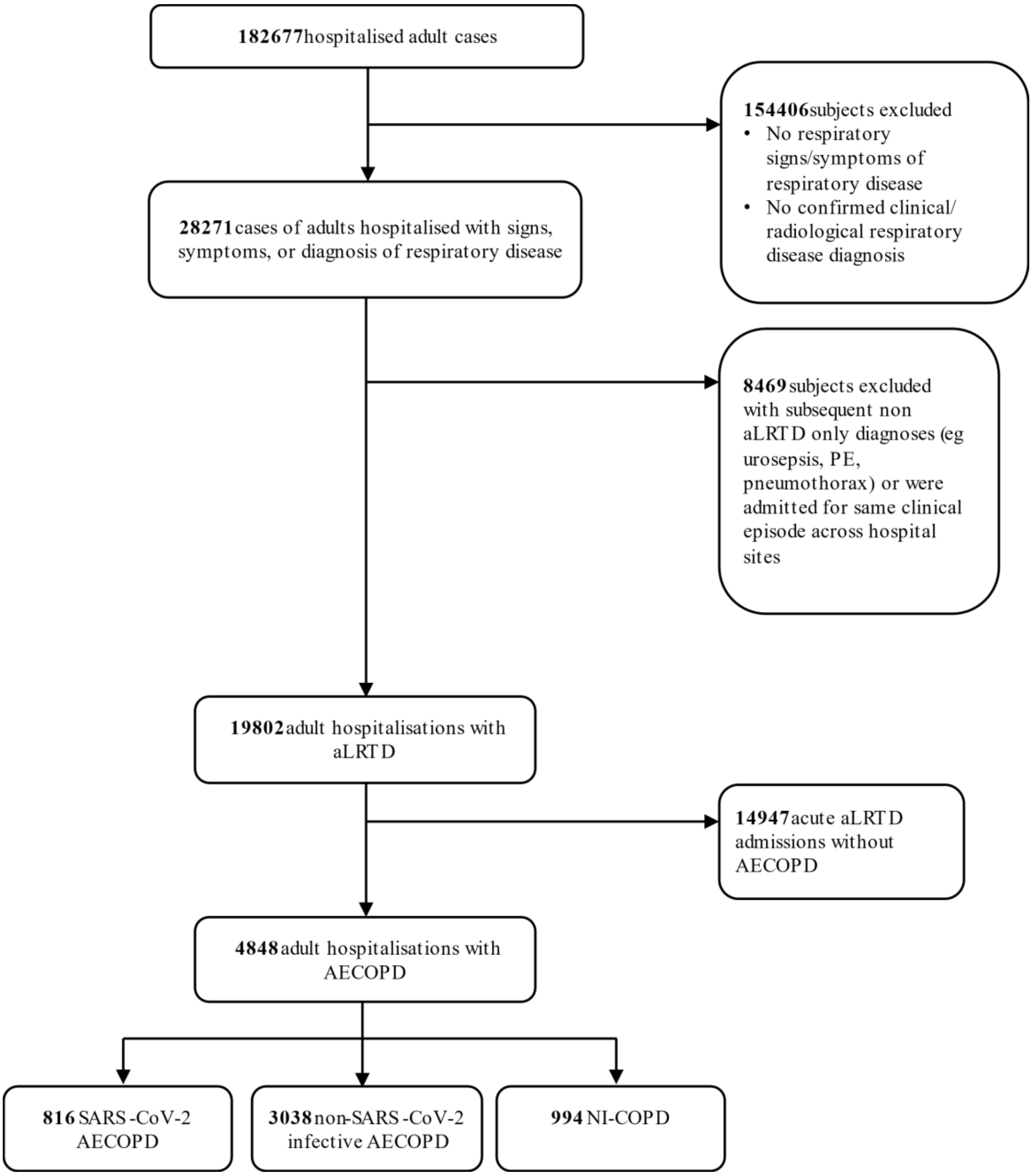

Supplementary Data 4: Patient characteristics and outcomes of individuals hospitalised with acute COPD exacerbations

**Characteristic**	**Overall**<br>**n = 4,848**	**Non-SARS-CoV-2 infective AECOPD**<br>**n = 3,038**	**SARS-CoV-2 AECOPD**<br>**n = 816**	**NICOPD**<br>**n = 994**	**p-value**
Age at admission					
Median (IQR)	75 (67, 82)	75 (66, 82)	75 (68, 83)	74 (67, 82)	0.048
Sex					0.8
Male	2,471 (51.0%)	1,552 (51.1%)	421 (51.6%)	498 (50.1%)	
Female	2,377 (49.0%)	1,486 (48.9%)	395 (48.4%)	496 (49.9%)	
Ethnicity					<0.001
White British	4,079 (84.1%)	2,591 (85.3%)	669 (82.0%)	819 (82.4%)	
White other	104 (2.1%)	58 (1.9%)	19 (2.3%)	27 (2.7%)	
Mixed origin	28 (0.6%)	16 (0.5%)	4 (0.5%)	8 (0.8%)	
Black	38 (0.8%)	18 (0.6%)	8 (1.0%)	12 (1.2%)	
Asian	35 (0.7%)	12 (0.4%)	17 (2.1%)	6 (0.6%)	
Other	23 (0.5%)	17 (0.6%)	5 (0.6%)	1 (0.1%)	
Unknown	541 (11.2%)	326 (10.7%)	94 (12%)	121 (12.2%)	
Care home resident					0.061
No	3,759 (77.5%)	2,364 (77.9%)	627 (76.8%)	768 (77.3%)	
Yes	344 (7.1%)	232 (7.6%)	60 (7.4%)	52 (5.2%)	
Unknown	745 (15.4%)	442 (14.5%)	129 (16.2%)	174 (17.7%)	
Smoking status					<0.001
Non-smoker	575 (11.9%)	352 (11.6%)	105 (12.8%)	118 (11.9%)	
Current smoker	991 (20.4%)	665 (21.9%)	114 (14.0%)	212 (21.3%)	
Ex-smoker	3,282 (67.7%)	2,021 (66.5%)	597 (73.2%)	664 (66.8%)	
COVID-19 vaccine doses					<0.001
Unvaccinated	1,793 (37.0%)	1,150 (37.9%)	337 (41.2%)	306 (30.8%)	
1 dose	569 (11.7%)	375 (12.3%)	67 (8.2%)	127 (12.8%)	
2 doses	1,416 (29.2%)	911 (30.0%)	183 (22.4%)	322 (32.5%)	
3 doses	972 (20.1%)	547 (18.0%)	206 (25.2%)	219 (22.1%)	
4 doses	96 (2.0%)	55 (1.8%)	23 (2.8%)	18 (1.8%)	
Unknown	2 (0.0%)	0 (0%)	0 (0%)	2 (0.2%)	
Pneumococcal Vaccine†					<0.001
Received PPV23	3,238 (66.8%)	1,985 (65.3%)	548 (67.2%)	705 (71.1%)	
Received PCV13	7 (0.1%)	1 (<0.1%)	3 (0.4%)	3 (0.3%)	
Not received	1,282 (26.5%)	862 (28.4%)	192 (23.5%)	228 (23.0%)	
Unknown	319 (6.6%)	190 (6.3%)	73 (8.9%)	56 (5.6%)	
Influenza Vaccine†					<0.001
Received	3,207 (66.2%)	1,959 (64.5%)	535 (65.6%)	713 (71.9%)	
Not received	1,348 (27.8%)	905 (29.8%)	211 (25.9%)	232 (23.4%)	
Unknown	291 (6.0%)	174 (5.7%)	70 (8.6%)	47 (4.7%)	
CCI					0.03
Not severe (<5)	1,756 (36.2%)	1,135 (37.4%)	264 (32.4%)	357 (35.9%)	
Severe (≥5)	3,092 (63.8%)	1,903 (62.6%)	552 (67.6%)	637 (64.1%)	
Rockwood frailty score					0.4
Rockwood <5	1,771 (36.5%)	1,120 (37.4%)	282 (34.6%)	369 (37.1%)	
Rockwood ≥5	3,077 (63.5%)	1,918 (62.6%)	534 (65.4%)	625 (62.9%)	

CURB-65 score					0.1
0 (Low risk)	735 (15.2%)	483 (15.9%)	105 (12.9%)	147 (14.8%)	
1 (Low risk)	2,630 (54.2%)	1,613 (53.1%)	443 (54.3%)	574 (57.7%)	
2 (Intermediate risk)	1,249 (25.8%)	786 (25.9%)	229 (28.1%)	234 (23.5%)	
3 (High risk)	212 (4.4%)	140 (4.6%)	36 (4.4%)	36 (3.6%)	
4 (High risk)	22 (0.5%)	16 (0.5%)	3 (0.4%)	3 (0.3%)	
Asthma					0.004
No	4,484 (92.5%)	2,828 (93.1%)	761 (93.3%)	895 (90.0%)	
Yes	364 (7.5%)	210 (6.9%)	55 (6.7%)	99 (10.0%)	
Bronchiectasis					0.001
No	4,535 (93.5%)	2,812 (92.6%)	778 (95.3%)	945 (95.1%)	
Yes	313 (6.5%)	226 (7.4%)	38 (4.7%)	49 (4.9%)	
IHD					0.006
No	3,978 (82.1%)	2,520 (83.9%)	638 (78.2%)	820 (82.5%)	
Yes	870 (17.9%)	518 (17.1%)	178 (21.8%)	174 (17.5%)	
Hypertension*					0.4
No	4,055 (83.6%)	2,550 (83.9%)	670 (82.1%)	835 (84.0%)	
Yes	793 (16.4%)	488 (16.1%)	146 (17.9%)	159 (16.0%)	
Congestive cardiac failure					<0.001
No	3,968 (81.8%)	2,543 (83.7%)	668 (81.9%)	757 (76.2%)	
Yes	880 (18.2%)	495 (16.3%)	148 (18.1%)	237 (23.8%)	
Immunodeficiency					<0.001
No	4,006 (82.6%)	2,489 (81.9%)	712 (87.3%)	805 (81.0%)	
Yes	842 (17.4%)	549 (18.1%)	104 (12.7%)	189 (19.0%)	
Diabetes mellitus					0.3
No	3,751 (77.4%)	2,361 (77.7%)	619 (75.9%)	771 (77.6%)	
Type 1	40 (0.8%)	30 (1.0%)	6 (0.7%)	4 (0.4%)	
Type 2	1057 (21.8%)	647 (21.3%)	191 (23.4%)	219 (22.0%)	
CKD**					0.057
No	3,647 (75.3%)	2,315 (76.2%)	602 (73.8%)	730 (73.6%)	
Mild	1,008 (20.8%)	608 (20.0%)	171 (21.0%)	229 (23.1%)	
Moderate/Severe	191 (3.9%)	115 (3.8%)	43 (5.3%)	33 (3.3%)	
Dementia					0.042
No	4,541 (93.7%)	2,849 (93.8%)	750 (91.9%)	942 (94.8%)	
Yes	307 (6.3%)	189 (6.2%)	66 (8.1%)	52 (5.2%)	
Cognitive impairment					0.5
No	4,620 (95.3%)	2,892 (95.2%)	774 (94.9%)	954 (96.0%)	
Yes	228 (4.7%)	146 (4.8%)	42 (5.1%)	40 (4.0%)	
CVA					0.4
No	4,480 (92.4%)	2,796 (92.0%)	758 (92.9%)	926 (93.2%)	
Yes	368 (7.6%)	242 (8.0%)	58 (7.1%)	68 (6.8%)	
TIA					0.8
No	4,593 (94.7%)	2,882 (94.9%)	769 (94.2%)	942 (95%)	
Yes	255 (5.3%)	156 (5.1%)	47 (5.8%)	52 (5.2%)	
Solid organ cancer					0.7
No	4,349 (89.7%)	2,717 (89.4%)	734 (90.0%)	898 (90.3%)	
Yes	499 (10.3%)	321 (10.6%)	82 (10.0%)	96 (9.7%)	
Leukaemia					0.7
No	4,808 (99.2%)	3,015 (99.2%)	809 (99.1%)	984 (99.0%)	
Yes	40 (0.8%)	23 (0.8%)	7 (0.9%)	10 (1.0%)	
Lymphoma					0.6

No	4,791 (98.8%)	3,006 (98.9%)	804 (98.5%)	981 (98.7%)	
Yes	57 (1.2%)	32 (1.1%)	12 (1.5%)	13 (1.3%)	
Taking inhaled corticosteroids					0.6
No	1,984 (40.9%)	1,229 (40.5%)	334 (41.0%)	421 (41.9%)	
Yes	2,856 (58.9%)	1,805 (59.5%)	480 (58.8%)	571 (57.9%)	
Unknown	8 (0.2%)	4 (0.1%)	2 (0.2%)	2 (0.2%)	
Pneumonia present					<0.001
No	2675 (55.2%)	1418 (46.7%)	298 (36.5%)	0 (0%)	
Yes	2173 (44.8%)	1620 (53.3%)	518 (63.4%)	994 (100%)	
30-day mortality					<0.001
Survived	4,314 (89.0%)	2,701 (88.9%)	678 (83.1%)	935 (94.1%)	
Died	534 (11.0%)	337 (11.1%)	138 (16.9%)	59 (5.9%)	
Length of hospital stay					<0.001
Median (IQR)	5 (2, 11)	5 (2, 10)	7 (3, 15)	4 (2, 9)	
Ventilatory support					<0.001
No	4,267 (88.0%)	2,683 (88.3%)	665 (81.5%)	919 (92.5%)	
Yes	581 (12.0%)	355 (11.7%)	151 (18.5%)	75 (7.5%)	

P-values represent the results of Kruskal-Wallis rank sum tests or Pearson's Chi-squared tests

*Hypertension was only included if causing other cardiac complications.
**Chronic kidney disease (CKD) was classified as mild if stage 1-3; moderate/severe if stage 4-5, end-stage renal failure or there was dialysis dependence.
†In the UK, all adults aged ≥65 years are eligible for Pneumococcal vaccination (PneumoVax®, PPV23) once, and annual influenza vaccine.

AECOPD, acute exacerbation of chronic obstructive pulmonary disease; PPV23, 23-valent pneumococcal polysaccharide vaccine; PCV13, 13-valent pneumococcal conjugate vaccine; IHD, ischaemic heart disease; CKD, chronic kidney disease; CVA, cerebrovascular accident; n, number; NICOPD, non-infective exacerbation of COPD; TIA, transient ischaemic attack; IQR, interquartile range.

Supplementary Data 5: Adjusted hazard ratios for logistic regression analysis of positive pressure ventilatory support

Characteristic	Group	Overall			Wild-type period			Alpha period			Delta period			Omicron period		
		n	aOR (95%CI)	p-value	n	aOR (95%CI)	p-value	n	aOR (95%CI)	p-value	n	aOR (95%CI)	p-value	n	aOR (95%CI)	p-value
Exacerbation type	Non-SARS-CoV-2 Infective AECOPD	3038	-	-	464	-	-	516	-	-	704	-	-	413	-	-
	SARS-CoV-2 AECOPD	816	1·55 (1·24-1·93)	<0·001	12	2·87 (0·52-12·07)	0·179	75	1·92 (0·91-3·90)	0·075	142	1·56 (0·91-2·60)	0·097	225	0·61 (0·35-1·07)	0·070
	NI-COPD	994	0·83 (0·63-1·09)	0·185	95	0·93 (0·36-2·10)	0·871	174	1·25 (0·66-2·28)	0·484	265	0·60 (0·30-1·10)	0·113	171	0·77 (0·41-1·38)	0·394
Age	-	4848	0·96 (0·95-0·97)	<0·001	574	0·94 (0·91-0·97)	<0·001	768	0·96 (0·93-0·98)	<0·001	1114	0·95 (0·93-0·98)	<0·001	812	0·97 (0·95-0·99)	0·011
CCI Score	0-4	1756	-	-	201	-	-	257	-	-	429	-	-	295	-	-
	≥5	3092	0·99 (0·79-1·25)	0·959	370	1·16 (0·56-2·45)	0·696	508	1·03 (0·56-1·92)	0·930	682	0·99 (0·59-1·67)	0·962	514	0·77 (0·44-1·36)	d
COVID vaccine	0 doses	1793	-	-	NA	NA	NA	204	-	-	118	-	-	95	-	-
	1 dose	569	0·96 (0·71-1·28)	0·777	NA	NA	NA	430	1·40 (0·78-2·58)	0·272	41	0·76 (0·24-2·12)	0·614	20	2·08 (0·51-7·28)	0·269
	2 doses	1416	0·80 (0·63-1·00)	0·053	NA	NA	NA	131	1·24 (0·52-2·88)	0·621	895	0·74 (0·41-1·36)	0·311	73	1·66 (0·66-4·21)	0·281
	3 doses	972	0·90 (0·70-1·16)	0·411	NA	NA	NA	NA	NA	NA	57	0·79 (0·21-2·44)	0·702	524	1·40 (0·70-3·01)	0·366
	4 doses	96	0·67 (0·29-1·34	0·297	NA	NA	NA	NA	NA	NA	NA	NA	NA	96	0·93 (0·33-2·55)	0·895
Smoking	No smoking history	575	-	-	130	-	-	73	-	-	85	-	-	129	-	-
	Current smoker	991	0·68 (0·49-0·95)	0·023	163	0·66 (0·30-1·49)	0·313	134	1.41 (0.53-4.20)	0·509	246	0·67 (0·27-1·79)	0·394	162	0·40 (0·19-0·83)	0·015
	Ex-smoker	3282	0·97 (0·74-1·29)	0·847	278	0·85 (0·42-1·78)	0·651	558	1.48 (0.65-4.04)	0·390	780	1·25 (0·56-3·14)	0·612	518	0·69 (0·39-1·26)	0·216
ICS	None	1992	-	-	209	-	-	331	-	-	433	-	-	348	-	-
	ICS	2856	0·98 (0·82-1·18)	0·861	362	0·69 (0·39-1·23)	0·202	434	1.13 (0.70-1.84)	0·620	678	1·16 (0·76-1·79)	0·498	461	0·97 (0·62-1·53)	0·900
Immune status	Immunocompetent	4006	-	-	546	-	-	616	-	-	823	-	-	683	-	-
	Immunodeficient	842	1·09 (0·86-1·39)	0·466	25	1·06 (0·23-3·43)	0·926	149	1.17 (0.63-2.08)	0·614	288	1·18 (0·73-1·86)	0·485	126	0·86 (0·45-1·55)	0·641
Sex	Male	2471	-	-	269	-	-	432	-	-	563	-	-	422	-	-
	Female	2377	0·90 (0·75-1·07)	0·230	302	0·97 (0·55-1·72)	0·913	333	0.80 (0.49-1.29)	0·370	548	1·02 (0·68-1·54)	0·916	387	0·89 (0·57-1·37)	0·596
ECAF score	0-1	3534	-	-	448	-	-	569	-	-	830	-	-	586	-	-
	2	1161	2·36 (1·93-2·88)	<0·001	106	2·06 (1·03-4·00)	0·037	179	2.83 (1.63-4.89)	<0·001	248	2·52 (1·60-3·97)	<0·001	201	1·50 (0·91-2·44)	0·104
	>2	153	4·77 (3·22-6·97)	<0·001	17	3·06 (0·62-11·21)	0·119	17	3.55 (0.76-12.32)	0·065	33	8·89 (3·92-19·89)	<0·001	22	3·17 (0·97-8·90)	0·038

AECOPD, acute exacerbation of COPD; CI, confidence interval; CCI, Charlson comorbidity index; ICS, inhaled corticosteroids; n, number; NA, not applicable; NI-COPD, non-infectious COPD exacerbation; aOR, adjusted Odds Ratio.

Supplementary Data 6: Adjusted hazard ratios for Cox Regression Analysis of hospital length of stay (LOS)

Characteristic	Group	Overall			Wild-type period			Alpha period			Delta period			Omicron period		
		n	aHR (95%CI)	p-value	n	aHR (95%CI)	p-value	n	aHR (95%CI)	p-value	n	aHR (95%CI)	p-value	n	aHR (95%CI)	p-value
Exacerbation type	Non-SARS-CoV-2 Infective AECOPD	3038	-	-	464	-	-	516	-	-	704	-	-	413	-	-
	SARS-CoV-2 AECOPD	816	1·26 (1·15-1·37)	<0·001	12	1·47 (0·80-2·7)	0·212	75	1·31 (0·97-1·8)	0·078	142	1·16 (0·95-1·4)	0·153	225	1·01 (0·84-1·2)	0·945
	NI-COPD	994	0·89 (0·83-0·96)	0·003	95	0·86 (0·58-1·1)	0·206	174	0·95 (0·79-1·1)	0·592	265	0·97 (0·84-1·1)	0·713	171	0·61 (0·50-0·75)	<0·001
COVID vaccine	0 doses	4848	-	-	NA	NA	NA	204	-	-	118	-	-	85	-	-
	1 dose	1756	0·98 (0·89-1·09)	0·766	NA	NA	NA	430	1·01 (0·84-1·2)	0·926	41	0·97 (0·65-1·4)	0·862	20	1·27 (0·75-2·17)	0·372
	2 doses	3092	0·94 (0·87-1·01)	0·102	NA	NA	NA	131	1·03 (0·80-1·3)	0·805	895	0·86 (0·70-1·1)	0·179	73	0·90 (0·65-1·25)	0·532
	3 doses	1793	0·97 (0·90-1·06)	0·546	NA	NA	NA	NA	NA	NA	57	0·80 (0·57-1·1)	0·214	524	0·78 (0·61-0·99)	0·044
	4 doses	569	0·44 (0·36-0·54)	<0·001	NA	NA	NA	NA	NA	NA	NA	NA	NA	96	0·18 (0·13-0·25)	<0·001
Age	-	1416	1·01 (1·01-1·01)	<0·001	574	1·00 (0·99-1·00)	0·621	768	1·01 (1·00-1·00)	0·003	1114	1·01 (1·00-1·00)	0·002	812	1·01 (1·00-1·02)	0·07
Sex	Male	972	-	-	269	-	-	432	-	-	563	-	-	422	-	-
	Female	96	0·95 (0·89-1·01)	0·085	302	1·05 (0·89-1·30)	0·557	333	0·97 (0·84-1·1)	0·728	548	0·89 (0·93-1·3)	0·0·73	387	0·87 (0·75-1·0)	0·057
CCI Score	0-4	575	-	-	201	-	-	257	-	-	429	-	-	295	-	-
	≥5	991	1·16 (1·07-1·25)	<0·001	370	1·52 (1·20-1·90)	<0·001	508	1·13 (0·92-1·4)	0·25	682	1·09 (0·93-1·3)	0·294	514	1·18 (0·97-1·43)	0·098
Smoking	No smoking history	3282	-	-	130	-	-	73	-	-	85	-	-	129	-	-
	Current smoker	1992	0·87 (0·78-0·97)	0·001	163	0·93 (0·72-1·20)	0·565	134	1·16 (0·86-1·6)	0·343	246	0·69 (0·53-0·9)	0·006	162	0·67 (0·51-0·87)	0·003
	Ex-smoker	2856	0·94 (0·85-1·03)	0·184	278	0·90 (0·72-1·10)	0·32	558	1·06 (0·83-1·4)	0·559	780	0·91 (0·72-1·2)	0·459	518	0·77 (0·62-0·96)	0·017
ICS	None	4006	-	-	209	-	-	331	-	-	443	-	-	348	-	-
	ICS	842	1·06 (1·00-1·13)	0·044	362	1·04 (0·66-1·20)	0·713	434	1·08 (0·93-1·3)	0·326	678	1·11 (0·98-1·3)	0·099	461	1·00 (0·86-1·16)	0·985
Immune status	Immunocompetent	2471	-	-	546	-	-	616	-	-	823	-	-	683	-	-
	Immunodeficient	2377	1·02 (0·94-1·10)	0·651	25	0·90 (0·58-1·40)	0·627	149	1·09 (0·90-1·3)	0·377	288	1·03 (0·89-1·2)	0·682	126	0·96 (0·78-1·17)	0·668
ECAF score	0-1	3534	-	-	448	-	-	569	-	-	830	-	-	201	-	-
	2	1161	1·30 (1·20-1·40)	<0·001	106	1·61 (1·28-2·00)	<0·001	179	1·21 (1·00-1·5)	0·051	248	1·40 (1·19-1·6)	<0·001	22	1·24 (1·0-·1·48)	0·02
	>2	153	1·52 (1·24-1·87)	<0·001	17	2·31 (1·34-4·00)	0·003	17	1·50 (0·74-3·0)	0·258	33	1·63 (1·01-2·6)	0·047	413	1·56 (0·93-2·64)	0·094

AECOPD, acute exacerbation of COPD; CI, confidence interval; CCI, Charlson comorbidity index; aHR, adjusted hazard ratio; ICS, inhaled corticosteroids; LOS, length of stay; n, number; NA, not applicable; NI-COPD, non-infectious COPD exacerbation

Supplementary Data 7: Adjusted hazard ratios for Cox Regression Analysis of 30-day mortality

Characteristic	Group	Overall			Wild-type period			Alpha period			Delta period			Omicron period		
		n	aHR (95%CI)	p-value	n	aHR (95%CI)	p-value	n	aHR (95%CI)	p-value	n	aHR (95%CI)	p-value	n	aHR (95%CI)	p-value
Exacerbation type	Non-SARS-CoV-2 Infective AECOPD	3038	-	-	464	-	-	516	-	-	704	-	-	413	-	-
	SARS-CoV-2 AECOPD	816	1·35 (1·10-1·65)	0·004	12	0·51 (0·07-3·78)	0·51	75	2·84 (1·61-5·0)	<0·001	142	1·16 (0·73-1·85)	0·534	225	0·61 (0·35-1·07)	0·083
	NI-COPD	994	0·75 (0·56-1·00)	0·046	95	0·23 (0·06-0·95)	0·042	174	1·26 (0·67-2·4)	0·468	265	0·81 (0·46-1·42)	0·452	171	0·68 (0·33-1·39)	0·285
Age	-	4848	1·03 (1·02-1·04)	<0·001	574	1·01 (0·980-1·04)	0·556	768	1·05 (1·02-1·1)	<0·001	1114	1·02 (1·00-1·05)	0·044	812	1·02 (0·99-1·05)	0·12
CCI Score	0-4	1756	-	-	201	-	-	257	-	-	429	-	-	295	-	-
	≥5	3092	1·58 (1·22-2·06)	<0·001	370	4·75 (1·63-13·80)	0·004	508	0·86 (0·45-1·7)	0·657	682	2·51 (1·39-4·54)	0·002	514	1·21 (0·62-2·34)	0·575
COVID vaccine	0 doses	1793	-	-	NA	NA	NA	204	-	-	118	-	-	95	-	-
	1 dose	569	0·79 (0·59-1·05)	0·11	NA	NA	NA	430	0·73 (0·42-1·3)	0·262	41	1·02 (0·43-2·42)	0·955	20	1·31 (0·36-1·36)	0·682
	2 doses	1416	0·74 (0·59-0·91)	0·005	NA	NA	NA	131	0·86 (0·42-1·8)	0·689	895	0·49 (0·28-0·85)	0·011	73	0·48 (0·15-1·54)	0·217
	3 doses	972	0·66 (0·513-0·84)	<0·001	NA	NA	NA	NA	NA	NA	57	0·32 (0·10-0·97)	0·044	524	0·69 (0·35-1·36)	0·282
	4 doses	96	0·21 (0·076-0·55)	0·002	NA	NA	NA	NA	NA	NA	NA	NA	NA	96	0·23 (0·07-0·75)	0·014
Smoking	No smoking history	575	-	-	130	-	-	73	-	-	85	-	-	129	-	-
	Current smoker	991	0·86 (0·61-1·22)	0·406	163	1·25 (0·56-2·80)	0·58	134	3·22 (1·03-10·0)	0·044	246	1·08 (0·43-2·66)	0·874	162	0·39 (0·16-0·99)	0·046
	Ex-smoker	3282	1·07 (0·82-1·40)	0·625	278	0·93 (0·48-1·80)	0·836	558	2·20 (0·79-6·1)	0·13	780	1·28 (0·58-2·80)	0·542	518	0·83 (0·46-1·50)	0·538
ICS	None	1992	-	-	209	-	-	331	-	-	433	-	-	348	-	-
	ICS	2856	1·56 (1·30-1·87)	<0·001	362	1·83 (0·97-3·46)	0·061	434	2·89 (1·72-4·9)	<0·001	678	1·51 (1·02-2·24)	0·039	461	0·58 (0·36-0·93)	0·024
Immune status	Immunocompetent	4006	-	-	546	-	-	616	-	-	823	-	-	683	-	-
	Immunodeficient	842	1·20 (0·94-1·52)	0·139	25	0·77 (0·18-3·27)	0·72	149	1·09 (0·60-2·0)	0·768	288	1·14 (0·75-1·76)	0·549	126	1·10 (0·56-2·18)	0·778
Sex	Male	2471	-	-	269	-	-	432	-	-	563	-	-	422	-	-
	Female	2377	0·81 (0·68-0·96)	0·016	302	1·14 (0·66-1·98)	0·64	333	0·78 (0·50-1·2)	0·269	548	0·79 (0·55-1·15)	0·22	387	0·79 (0·49-1·27)	0·331
ECAF score	0-1	3534	-	-	448	-	-	569	-	-	830	-	-	586	-	-
	2	1161	2·52 (2·08-3·04)	<0·001	106	2·14 (1·16-3·95)	0·014	179	2·24 (1·35-3·7)	0·002	248	2·86 (1·89-4·33)	<0·001	201	2·20 (1·32-3·66)	0·003
	>2	153	6·04 (4·54-8·03)	<0·001	17	3·68 (1·40-9·69)	0·008	17	8·60 (4·06-18·2)	<0·001	33	8·20 (4·61-14·58)	<0·001	22	6·86 (3·10-15·19)	<0·001

AECOPD, acute exacerbation of COPD; CI, confidence interval; CCI, Charlson comorbidity index; aHR, adjusted hazard ratio; ICS, inhaled corticosteroids; n, number; NA, not applicable; NI-COPD, non-infectious COPD exacerbation

Supplementary Data 8: Patient characteristics and outcomes of individuals hospitalised with acute COPD exacerbations, divided by inhaled corticosteroid use

Characteristic	Overall<br>n = 4,840	Not taking inhaled corticosteroids<br>n = 1,984	Taking inhaled corticosteroids<br>n = 2,856	p-value
Exacerbation type				0.6
Non-SARS-CoV-2 Infective AECOPD	3,034 (62.7%)	1,229 (61.9%)	1,805 (63.2%)	
SARS-CoV-2 AE COPD	814 (16.8%)	334 (16.8%)	480 (16.8%)	
NI COPD	992 (20.5%)	421 (21.2%)	571 (20.0%)	
Age at admission				0.7
Median (IQR)	75 (67, 82)	75 (66, 82)	75 (67, 82)	
Sex				0.7
Male	2,463 (50.9%)	1,003 (50.6%)	1,460 (51.1%)	
Female	2,377 (49.1%)	981 (49.4%)	1,396 (48.9%)	
Ethnicity				0.8
White British	4,075 (84.2%)	1,675 (84.4%)	2,400 (84.0%)	
White other	104 (2.1%)	45 (2.3%)	59 (2.1%)	
Mixed origin	28 (0.6%)	13 (0.7%)	15 (0.5%)	
Black	38 (0.8%)	15 (0.8%)	23 (0.8%)	
Asian	35 (0.7%)	13 (0.7%)	22 (0.8%)	
Other	23 (0.5%)	12 (0.6%)	11 (0.4%)	
Unknown	537 (11.1%)	211 (10.6%)	326 (11.4%)	
Care home resident				0.8
No	3,751 (78%)	1,559 (79%)	2,192 (77%)	
Yes	344 (7.1%)	146 (7.4%)	198 (6.9%)	
Unknown	745 (15%)	279 (14%)	466 (16%)	
Smoking status				0.016
Non-smoker	574 (11.9%)	253 (12.8%)	321 (11.2%)	
Current smoker	990 (20.5%)	434 (21.9%)	556 (19.5%)	
Ex-smoker	3,276 (67.7%)	1,297 (65.4%)	1,979 (69.3%)	
COVID-19 vaccine doses				<0.001
Unvaccinated	1,792 (37.0%)	727 (36.6%)	1,065 (37.3%)	
1 dose	567 (11.7%)	275 (13.9%)	292 (10.2%)	
2 doses	1,416 (29.3%)	597 (30.1%)	819 (28.7%)	
3 doses	967 (20.0%)	351 (17.7%)	616 (21.6%)	
4 doses	96 (2.0%)	34 (1.7%)	62 (2.2%)	
Pneumococcal Vaccine†				0.2
Received PPV23	3,233 (66.8%)	1,315 (66.3%)	1,918 (67.2%)	
Received PCV13	7 (0.1%)	2 (0.1%)	5 (0.2%)	
Not received	1,280 (26.5%)	549 (27.7%)	731 (25.6%)	
Unknown	318 (6.6%)	118 (5.9%)	200 (7.0%)	
Influenza Vaccine†				0.071
Received	3,202 (66.2%)	1,316 (66.3%)	1,886 (66.1%)	
Not received	1,346 (27.8%)	567 (28.6%)	779 (27.3%)	
Unknown	290 (6.0%)	101 (5.1%)	189 (6.6%)	

Charlson comorbidity index				>0.9
Not severe (<5)	1,753 (36.2%)	721 (36.3%)	1,032 (36.1%)	
Severe (≥5)	3,087 (63.8%)	1,263 (63.7%)	1,824 (63.9%)	
Rockwood frailty score				0.005
Rockwood <5	1,768 (36.5%)	771 (38.9%)	997 (34.9%)	
Rockwood ≥5	3,072 (63.5%)	1,213 (61.1%)	1,859 (65.1%)	
CURB-65 score				0.2
0 (Low risk)	735 (15.2%)	299 (15.1%)	436 (15.3%)	
1 (Low risk)	2,624 (54.2%)	1,099 (55.4%)	1,525 (53.4%)	
2 (Intermediate risk)	1,247 (25.8%)	502 (25.3%)	745 (26.1%)	
3 (High risk)	212 (4.4%)	79 (4.0%)	133 (4.7%)	
4 (High risk)	22 (0.5%)	5 (0.3%)	17 (0.6%)	
Asthma				0.6
No	4,477 (92.5%)	1,830 (92.2%)	2,647 (93.7%)	
Yes	363 (7.5%)	154 (7.8%)	209 (7.3%)	
Bronchiectasis				>0.9
No	4,527 (93.5%)	1,855 (93.5%)	2,672 (93.6%)	
Yes	313 (6.5%)	129 (6.5%)	184 (6.4%)	
IHD				0.7
No	3,973 (82.1%)	1,634 (82.4%)	2,339 (81.9%)	
Yes	867 (17.9%)	350 (17.6%)	517 (18.1%)	
Hypertension*				0.06
No	4,048 (83.6%)	1,635 (82.4%)	2,413 (84.5%)	
Yes	792 (16.4%)	349 (17.6%)	443 (15.5%)	
Congestive cardiac failure				0.033
No	3,961 (81.8%)	1,595 (80.4%)	2,366 (82.8%)	
Yes	879 (18.2%)	389 (19.6%)	490 (17.2%)	
Immunodeficiency				0.8
No	3,999 (82.6%)	1,635 (82.4%)	2,364 (82.8%)	
Yes	841 (17.4%)	349 (17.6%)	492 (17.2%)	
Diabetes mellitus				0.6
None	3,743 (77.2%)	1,520 (77.6%)	2,223 (77.8%)	
Type 1	40 (0.8%)	16 (0.8%)	24 (0.8%)	
Type 2	1,057 (22.8%)	448 (22.6%)	609 (21.3%)	
CKD**				0.2
No	3,640 (75.2%)	1,478 (74.5%)	2,162 (75.8%)	
Mild	1,007 (20.8%)	417 (21.0%)	590 (20.7%)	
Moderate/Severe	191 (3.9%)	89 (4.5%)	102 (3.6%)	
Dementia				0.9
No	4,533 (93.7%)	1,860 (93.8%)	2,673 (93.6%)	
Yes	307 (6.3%)	124 (6.2%)	183 (6.4%)	
Cognitive impairment				0.5
No	4,613 (95.3%)	1,885 (95.0%)	2,728 (95.5%)	
Yes	227 (4.7%)	99 (5.0%)	128 (4.5%)	
CVA				0.6
No	4,472 (92.4%)	1,838 (92.6%)	2,634 (92.2%)	

Yes	368 (7.6%)	146 (7.4%)	222 (7.8%)	
TIA				0.8
No	4,585 (94.7%)	1,877 (94.6%)	2,708 (94.8%)	
Yes	255 (5.3%)	107 (5.4%)	148 (5.2%)	
Solid organ cancer				0.4
No	4,342 (89.7%)	1,790 (90.2%)	2,552 (89.4%)	
Yes	498 (10.3%)	194 (9.8%)	304 (10.6%)	
Leukaemia				>0.9
No	4,800 (99.2%)	1,967 (99.1%)	2,833 (99.2%)	
Yes	40 (0.8%)	17 (0.9%)	23 (0.8%)	
Lymphoma				0.3
No	4,783 (98.8%)	1,965 (99.0%)	2,818 (98.7%)	
Yes	57 (1.2%)	19 (1.0%)	38 (1.3%)	
30-day mortality				<0.001
Survived	4,307 (89.0%)	1,824 (91.9%)	2,483 (86.9%)	
Died	533 (11.0%)	160 (8.1%)	373 (13.1%)	
Length of hospital stay	5 (2, 11)	5 (2, 10)	5 (2, 11)	0.12
Median (IQR)				0.5
Ventilatory support	4,261 (88.0%)	1,755 (88.5%)	2,506 (87.7%)	
No	579 (12.0%)	229 (11.5%)	350 (12.3%)	
Yes				0.7

8 individuals were excluded from this table because their inhaled corticosteroid use was unknown

Pearson's Chi-squared test; Kruskal-Wallis rank sum test
*Hypertension was only included if causing other cardiac complications.
**Chronic kidney disease (CKD) was classified as mild if stage 1-3; moderate/severe if stage 4-5, end-stage renal failure or there was dialysis dependence.
†In the UK, all individuals aged ≥65 years are eligible for Pneumococcal vaccination (PneumoVax®, PPV23) once, and annual influenza vaccine.

AECOPD, acute exacerbation of chronic obstructive pulmonary disease; CKD, chronic kidney disease; CVA, cerebrovascular accident; IHD, ischaemic heart disease; IQR, interquartile range; n, number; NICOPD, non-infective exacerbation of COPD; PPV23, 23-valent pneumococcal polysaccharide vaccine; PCV13, 13-valent pneumococcal conjugate vaccine; TIA, transient ischaemic attack.

Supplementary Data 9: Regression analyses after optimal matching on propensity scores

Plots of (A) adjusted Odds Ratio after logistic regression analysis of requirement of positive pressure ventilatory support, (B) adjusted Hazard Ratio after Cox regression analysis of hospital length of stay and (C) 30-day mortality in patients hospitalised with COPD exacerbations after optimal matching on propensity scores

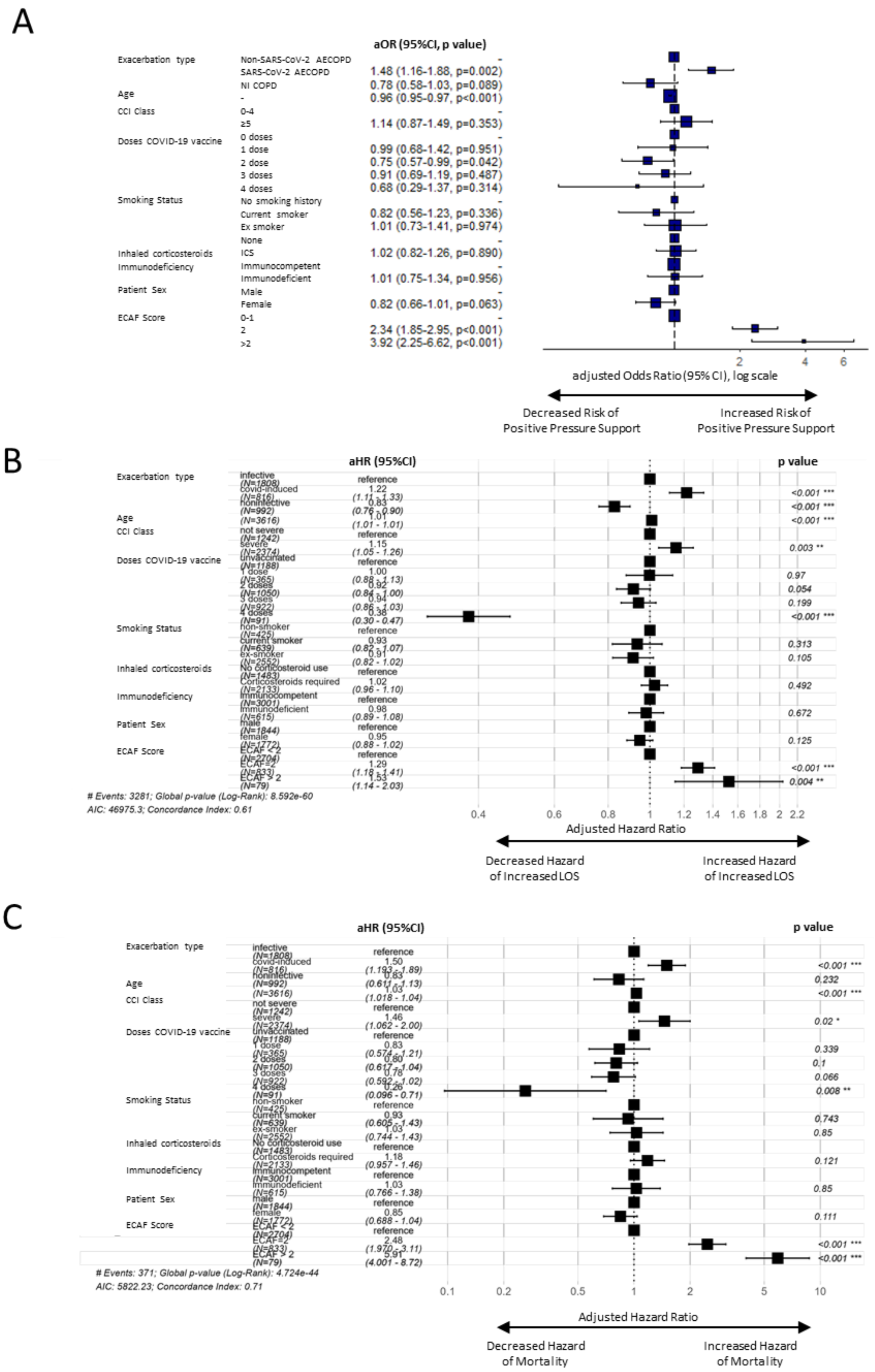

Supplementary Data 10: Subgroup Analysis of positive pressure support after stratification by patient sex

Plots of (A) adjusted Odds Ratio after logistic regression analysis of requirement of positive pressure ventilatory support for the (A) female and (B) male subgroups

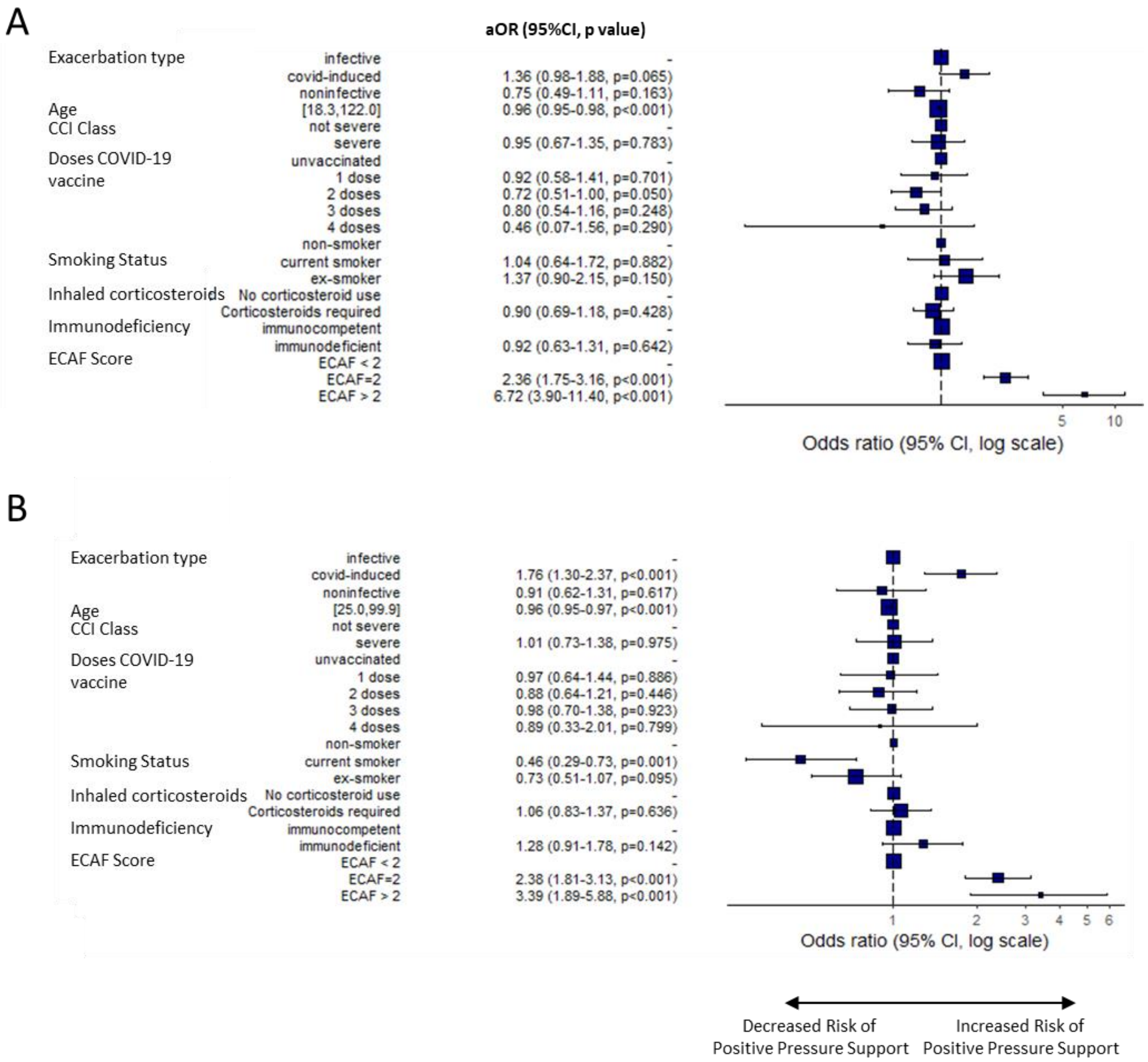

Supplementary Data 11: Subgroup Analysis of hospital length of stay after stratification by patient sex

Plots of adjusted Hazards Ratio after Cox regression analysis of hospital length of stay for the (A) female and (B) male subgroups

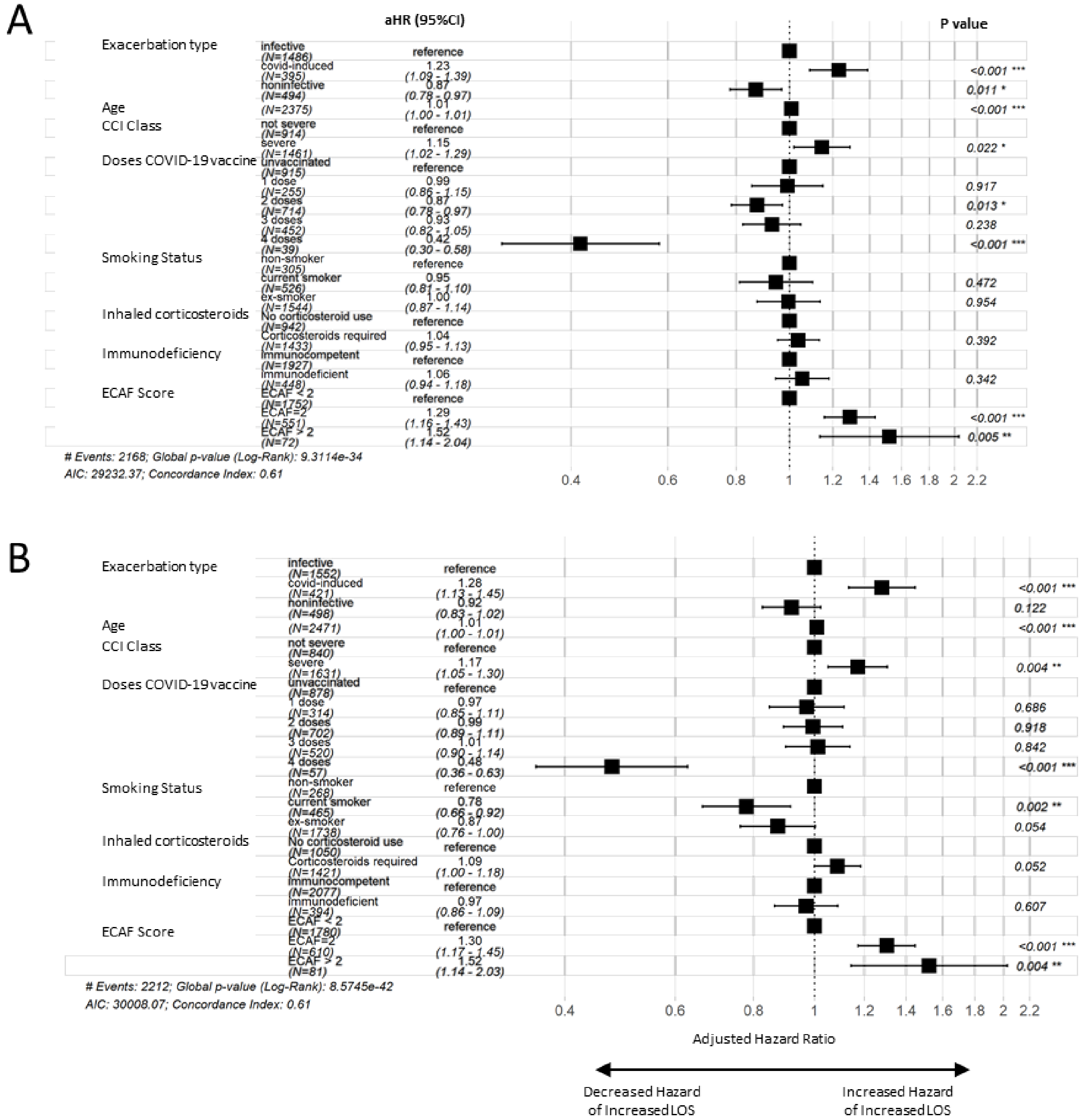

Supplementary Data 12: Subgroup Analysis of 30-day mortality after stratification by patient sex

Plots of adjusted Hazards Ratio after Cox regression analysis of 30-day mortality in patients hospitalised with COPD exacerbations for the (A) female and (B) male subgroups.

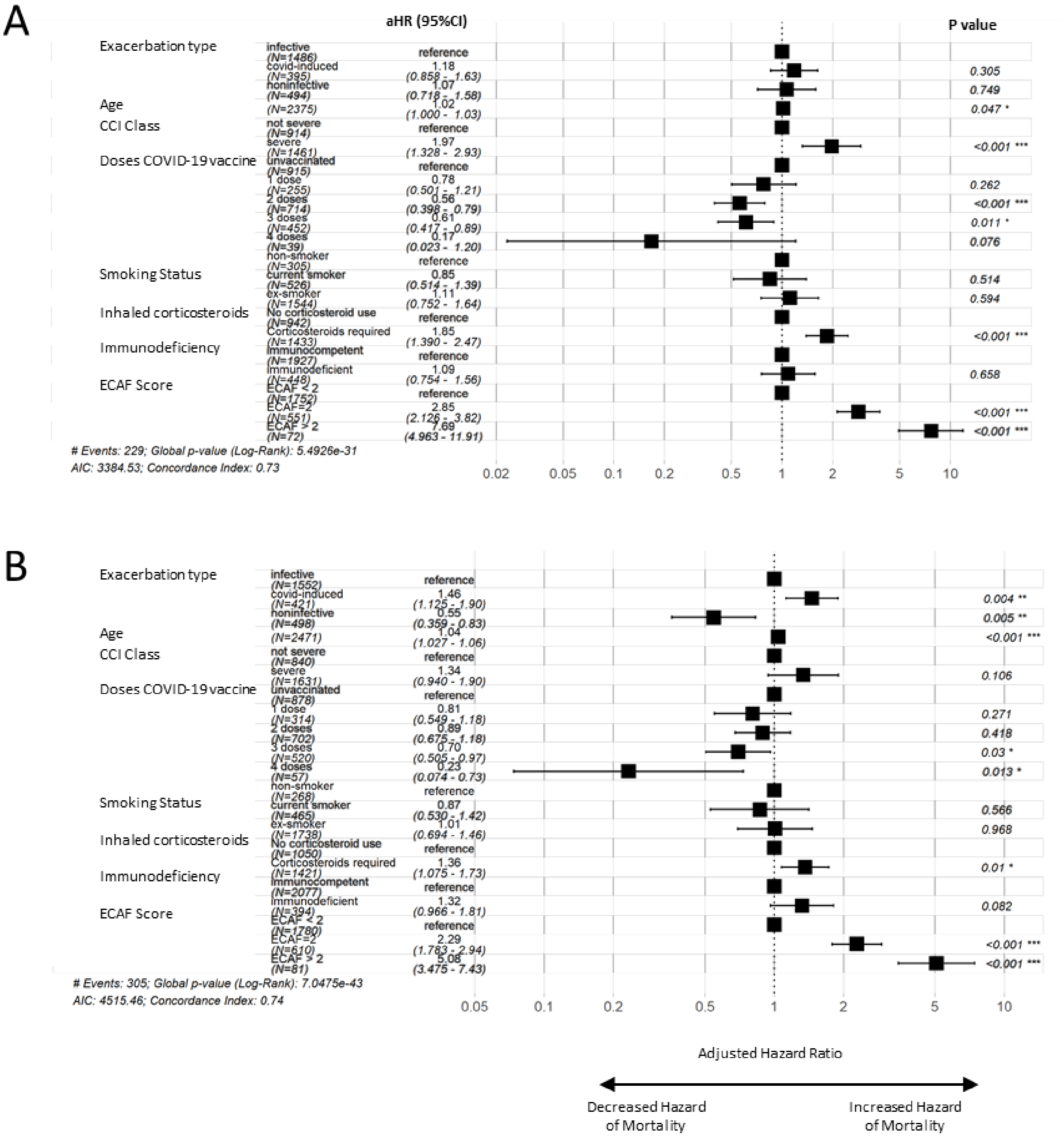

Supplementary Data 13: Subgroup Analysis of positive pressure support after stratification by age

Plots of (A) adjusted Odds Ratio after logistic regression analysis of requirement of positive pressure ventilatory support for the (A) under 65 and (B) over 65 subgroups

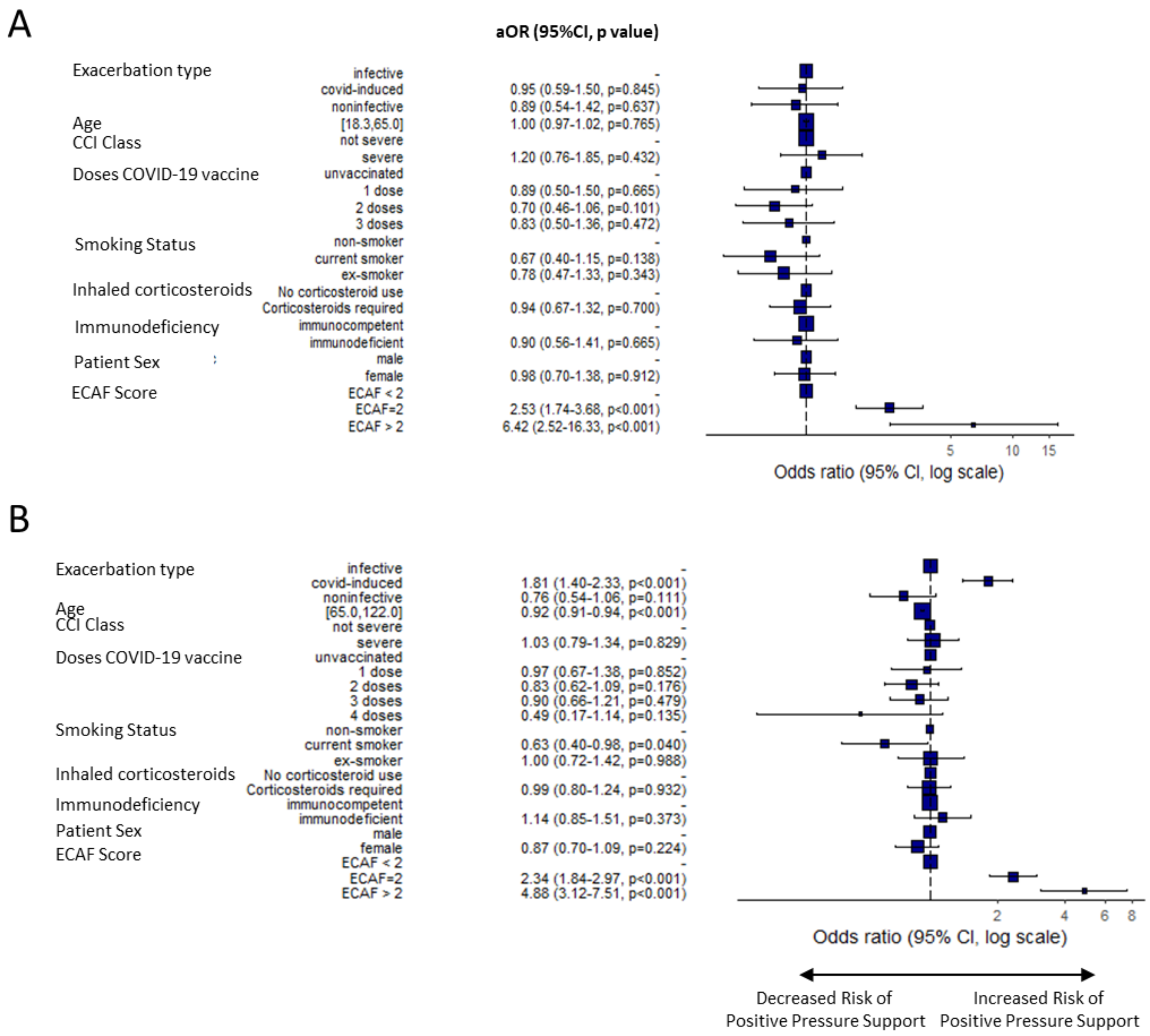

Supplementary Data 14: Subgroup Analysis of hospital length of stay after stratification by age

Plots of adjusted Hazards Ratio after Cox regression analysis of hospital length of stay for the (A) under 65 and (B) over 65 subgroups

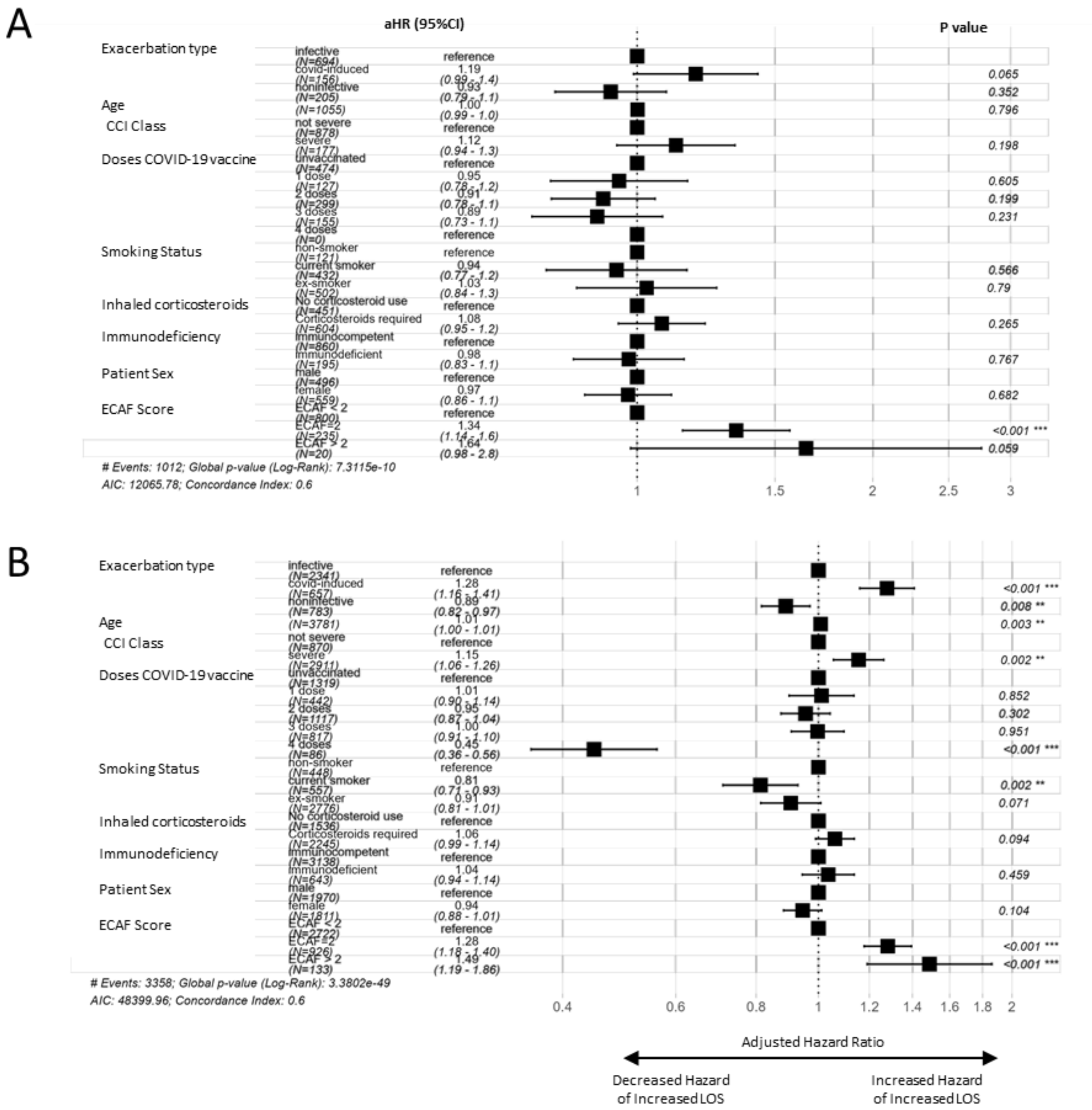

Supplementary Data 15: Subgroup Analysis of 30-day mortality after stratification by age

Plots of adjusted Hazards Ratio after Cox regression analysis of 30-day mortality in patients hospitalised with COPD exacerbations for the (A) under 65 and (B) over 65 subgroups.

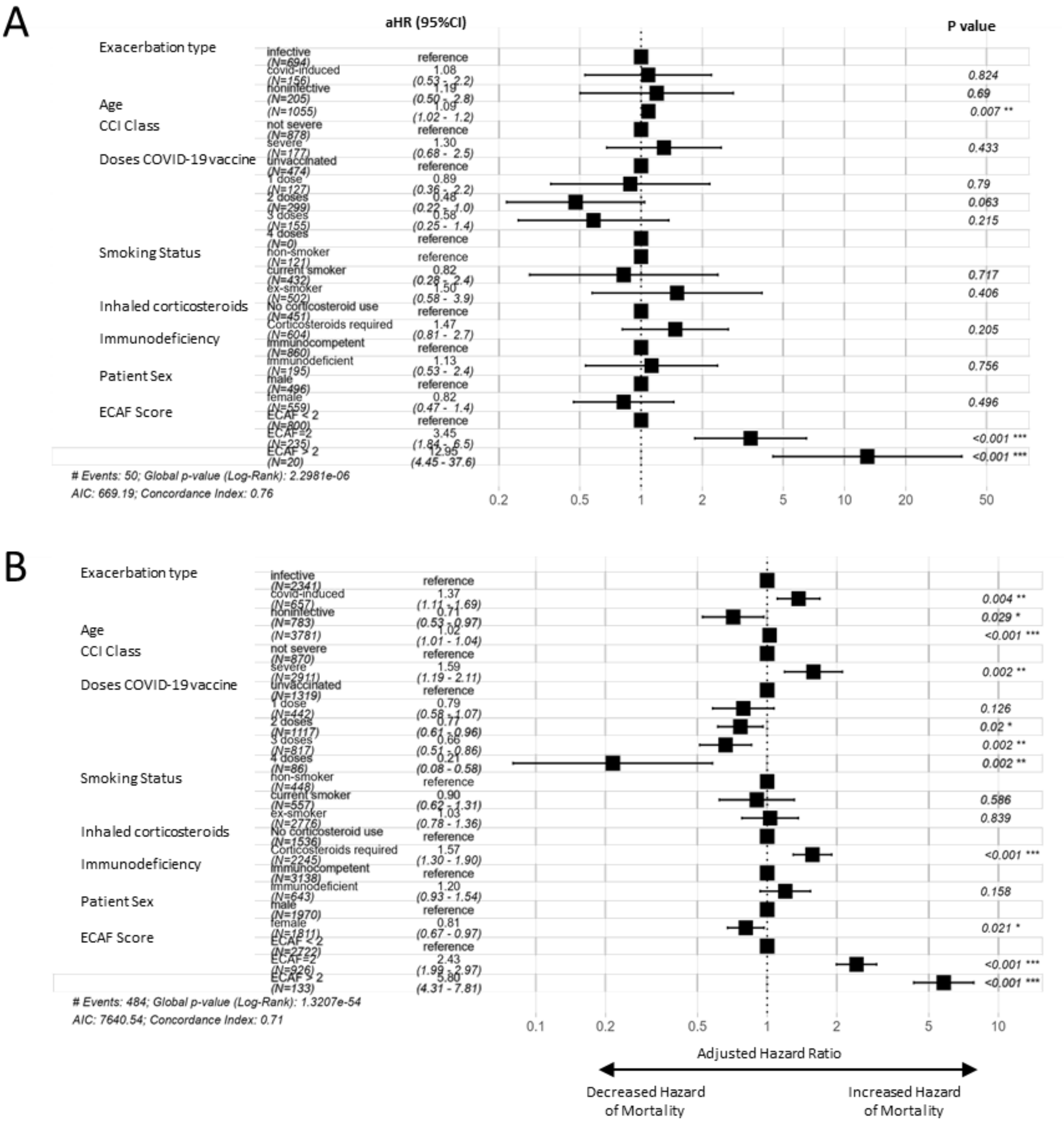

Supplement: sj-pdf-4-jrs-10.1177_01410768231184162 - Supplemental material for Impact of SARS-CoV-2 infective exacerbation of chronic obstructive pulmonary disease on clinical outcomes in a prospective cohort study of hospitalised adults [file sj-pdf-4-jrs-10.1177_01410768231184162.pdf]
