# Supplementary material for: Impact of SARS-CoV-2 infective exacerbation of chronic obstructive pulmonary disease on clinical outcomes in a prospective cohort study of hospitalised adults
Source: J R Soc Med. 2023 Jul 5;116(11):371–85. doi: 10.1177/01410768231184162 (PMC10686205; doi:10.1177/01410768231184162)
Supplement: sj-pdf-1-jrs-10.1177_01410768231184162 - Supplemental material for Impact of SARS-CoV-2 infective exacerbation of chronic obstructive pulmonary disease on clinical outcomes in a prospective cohort study of hospitalised adults [file sj-pdf-1-jrs-10.1177_01410768231184162.pdf]

Bristol Vaccine Centre
University of Bristol
Level 6, UHBW Education & Research Centre,
Upper Maudlin St,
Bristol, BS2 8AE
Tel: 0117 342 0172

Dear Editor,

Re: Manuscript ID JRSM-23-0010

"Impact of SARS-CoV-2 infective exacerbation of chronic obstructive pulmonary disease (COPD) on clinical outcomes in a prospective cohort study of hospitalised adults" which you submitted to the Journal of the Royal Society of Medicine

We would like to take the opportunity to thank the Editor and Reviewer for their time and effort in providing suggestions on how to improve the manuscript and analyses.

The original reviewer comments did not include those from Reviewer 1, and so we only responded to Reviewer 2. The Editor subsequently forwarded these missing comments on to us, and we have addressed them. Meantime we understand that our original response has been forwarded to Reviewer 2 (Statistics expert). Please note that some of the original responses have now been amended following the new comments from Reviewer 1. Accordingly, we have updated the track changes from the first response to provide a single version covering both reviewers comments.

We think that the changes we have made have significantly improved the manuscript.

We are grateful to you for giving the paper further consideration.

Yours sincerely

Adam Finn
Professor of Paediatrics, University of Bristol
Honorary Consultant, Bristol Royal Hospital for Children

Responses to comments: *Manuscript ID JRSM-23-0010*

The line numbers mentioned in the editors' and reviewers' comments are those created in the generated pdf of the original submission – the line numbers mentioned in the responses are those in the "changes accepted" resubmitted version

New text additions are shown in red, with deleted text shown in strike-through.

Reviewer: 1

1. There is no comment on the microbiological pneumonia type (bacterial vs. viral vs. fungal etc.) as well as the specific causal organism. Did this impact results?

Thank you. In this analysis we attempted to determine if exacerbation with SARS-CoV-2 is more severe than exacerbation due to other pathogens (all cause) and non-infectious aetiologies. We therefore did not split the 'non-COVID-19 infective exacerbation' cohort by pathogen and we acknowledge, in the paper, that there was little standard of care testing (excepting COVID-19 tests) during the time period observed. We have amended the existing comment on this matter in the limitations section (line 345-349), so that this now reads:

> *'Additionally, to prioritise SARS-CoV-2 testing, study hospitals undertook limited microbiological testing for other respiratory pathogens and, using standard-of-care results, while we are unable to comment on the microbial aetiology of non-SARS-CoV-2 respiratory infection we acknowledge that different pathogens may be associated with different severities.'*

2. Authors need to let readers to know how they deal with missing data.

Thank you – there were very few missing data, and as such we simply omitted missing data points. At the end of the methods section (line 237), we have now added:

> *'Missing data points were omitted from the analyses'*

3. Outcomes were not adjudicated - I would expect some outcome ascertainment process if this was a prospective cohort study.

Thank you – we selected three outcomes (1) 30-day mortality (2) length of hospital admission and (3) positive pressure support. These are all clinically relevant markers of severity and/or are important in resource allocation and healthcare planning to ensure appropriate care for patients with COPD. These outcomes are not subjective – patients either died within 30 days of hospitalisation or survived, had a defined admission/discharge date (and therefore admission length) and were either prescribed positive pressure ventilatory support or not – as such we do not believe that adjudication is required.

4. Prescription records of key medications, including angiotensin-converting enzyme inhibitor and/or angiotensin receptor blocker, β-blockers, calcium channel blockers, diuretics, lipid-lowering agents, antiplatelets, nonsteroidal anti-inflammatory drugs and insulin are not recorded or reported, so it is uncertain whether the differences observed are accounted for by differences in characteristics. In addition, baseline laboratory test results are not documented.

Thank you. There were minimal differences between the rates of IHD, hypertension, congestive cardiac failure and diabetes mellitus found in each of the three groups analysed in the cohort. As these patients are all managed by clinicians within a defined geographical

area, including the same specialist secondary care services, we think that it is unlikely that the rates of medication prescription and adherence would vary significantly between the three groups.

However, we did not collect these data and acknowledge that unexpected differences may be found in medications prescribed (noting that there was no difference in rate of inhaled corticosteroid prescription between the three groups) and therefore we have added the following text (line 342):

> *'We did not collect data on medication prescription or adherence, with the exception of inhaled corticosteroids, and thus cannot adjust by use of medication such as beta-blockers or angiotensin-converting enzyme inhibitor.'*

We deliberately did not adjust by baseline laboratory results, since we would expect patients who were immunodeficient or older to have weaker immune responses (e.g. lower white cell count and lower C-reactive protein) and therefore this would have been included in the model twice. Further, there is variability in time from symptom onset until presentation, which would affect the time of first test and thus the phase/severity of the immune response and tests measured.

5. The propensity score matching approach used is unclear. How were the propensity scores of each patient in the cohort with the confounding variables calculated? How was the balance in baseline covariates between groups evaluated? Show baseline and clinical characteristics of the study cohort before and after propensity score matching and distributions of propensity scores.

Thank you. Part of this is covered by our response to Reviewer 2 in their second comment, in which we provided additional text to clarify the propensity score calculations (line 233):

> *'Finally, we also performed propensity score matching as a sensitivity analysis; logistic regression on all covariates is used to define propensity scores, followed by matching using optimal pair matching ~~by matching on all c ovariates based on propensity scores~~.'*

We also show propensity scores before and after matching in Supplementary Data 2); clinical outcomes remain consistent after the analysis (Supplementary Figure 9).

6. Sensitivity analyses based on different propensity score matching approaches, including propensity score stratification, high-dimensional propensity score (HDPS) matching, and inverse probability of treatment weighting (IPTW) with consistent results would help validate the study findings.

Thank you for this suggestion – the propensity score matched analysis was performed only as a sensitivity analysis. The authors agree that it would be interesting to use several different approaches but feel that a single, clearly-documented method (optimal matching

on propensity scores) illustrate the message from this analysis more clearly, and thus prefer not to undertake these additional analyses.

7. I suggest subgroup analyses by age stratification and sex.

Thank you. We repeated the analysis by stratifying on age (with under and over 65-year-olds being the two groups), as well as sex. We found that the effect of COVID-induced exacerbations on all three clinical outcomes was statistically significant for males, but not females, apart from length of hospitalisation, as well as for patients over 65 years of age, but not under 65. We have therefore written (lines 232 and 284):

> *'We also carried out subgroup analyses by age stratification (namely, patients >65 years and <65) and sex.'*
>
> *and*
>
> *'From the subgroup analyses stratified by age and sex, we found that the effect of COVID-induced exacerbations on all three clinical outcomes was statistically significant for males, but not females (apart from length of hospitalisation), as well as for patients over 65 years of age, but not under 65 (Supplementary Data 10-15)'*

8. The rationale for including only a selection of confounders in the regression models is unclear.

Thank you. In line with appropriate statistical methodology, confounders were decided 'a priori' and not after we undertook a univariate analysis. We have added the following text (line 225):

> *'These confounders were decided a priori.'*

9. How the authors selected the dates used in the study to restrict analysis to periods of time when each variant was dominant in circulation is unclear.

Thank you. We included a supplementary figure (Supplementary Data 1) with data from the Wellcome Sanger Institute COVID-19 Genomic Surveillance which shows the dominant variant of concern and time periods we used to restrict these analyses. In the manuscript, we refer to this on line 227:

> *'We performed a sensitivity analysis, restricting analyses to time-periods when specific VOCs dominated (Supplementary-Data 1), thereby adjusting for time-dependent variables including healthcare resource usage and demand, public health*

interventions, available COVID-19 therapies, COVID-19 vaccination programme stage, and severity of the dominant variant.'

<u>Reviewer: 2</u>

1. The use of Cox regression analysis for the outcomes 'ventilation' and '30-day mortality' is correct. However, why is it used for 'length of hospital stay' (a continuous variable)? This type of analysis is appropriate for dichotomous variables only.

The 'ventilation' outcome is now referred to as 'positive pressure support' (as per comment 6), and we analysed this using a logistic regression model (stated on line 219):

> *'For analysis of ~~ventilatory support~~ positive pressure support, we used a logistic regression model.'*

We agree with the Reviewer that Cox regression analysis is only appropriate to use when there is a dichotomous variable. We used Cox regression analysis of the outcome '30-day mortality' and 'length of hospital stay' as they are both forms of survival analyses. For the hospital length of stay, the dichotomy is discharged versus not discharged and for mortality the dichotomy is alive versus dead. Hence, a Cox regression for 'hospital stay' estimates the increase in hazard ratio of a patient not being discharged. For instance, Figure 3a shows that having a SARS-CoV2 AECOPD is associated with a 26% increased risk of remaining in hospital compared to Non-SARS-CoV2 AECOPD.

The original manuscript was not clear on this point, and we thank the reviewer for highlighting this to us and allowing us to update and improve the manuscript. In response, we have provided additional text (line 217):

> *'Cox proportional regression analysis of length of hospital stay was used as this estimated the increase in hazard ratio of a patient not being discharged and, thus, having a longer stay in hospital.'*

2. A matched analysis is said to have been performed using propensity scores. More details need to be provided on the type of propensity analysis used and the matching process.

Thank you – following comments from reviewer one which we received after our initial reply, we have now amended the text on line 233:

'Finally, we also performed propensity score matching as a sensitivity analysis; logistic regression on all covariates is used to define propensity scores, followed by matching using optimal pair matching ~~by matching on all covariates based on propensity scores~~.'

3. It would be useful to have the flowchart showing the various subgroups of patients in the main paper (rather than as a supplementary figure).

We agree that it is important to have a flow diagram showing the subgroups of patients to aid the reader (and report in keeping with CONSORT guidelines). However, there are already 4 large multi-panel figures and 1 table, and the journal submission policy has a limit of 5 tables/figures in the main paper.

We have therefore included this as a supplementary figure and specified the numbers in the manuscript (line 242).

The authors would be happy if the Editor decided to include the flow diagram as an additional main figure, although this would take the submission over the journal's specifications.

4. Table 1 currently shows both baseline characteristics and outcomes. It would seem more appropriate to have separate tables for these two types of information.

Thank you – as highlighted above, the journal has a limited of five tables and figures for this style of article and we regret that we would be over this limit where we to split this table. We have divided the table rows into sections (Demographics, Risk Scores, Comorbidities, Patient Outcomes) to make this clearer for the reader.

5. Figures 3 and 4 have misaligned category labels for the hazard ratios.

Thank you – we have changed the category labels for the hazard ratios and updated the figures in the submission.

6. The use of the two terms 'ventilation support' and 'positive pressure support' in the text (which describe the same outcome) is confusing.

Thank you. We have amended the term 'ventilation support' to 'positive pressure support' where appropriate throughout the manuscript.

<u>Editorial comments (for a paper):</u>

1. Provide a competing interests statement for all authors.

Done – we include a competing interests statement (line 472):

'CH is Principal Investigator of the AvonCAP study which is an investigator-led University of Bristol study funded by Pfizer and has previously received support from the NIHR in an Academic Clinical Fellowship. JO is a Co-Investigator on the AvonCAP Study. LD is further supported by UKRI through the JUNIPER consortium (grant number MR/V038613/1), MRC (grant number MC/PC/19067), EPSRC (EP/V051555/1). AF is a member of the UK Joint Committee on Vaccination and Immunization (JCVI) and was, until December 2022, chair of the World Health Organization European Technical Advisory Group of Experts on Immunization (ETAGE) committee. In addition to his employers receiving funding from Pfizer as Chief Investigator of this study, he leads another project investigating transmission of respiratory bacteria in families jointly funded by Pfizer and the Gates Foundation and is an investigator in trials of COVID19 vaccines including ChAdOx1nCOV-19, Janssen and Valneva vaccines. EB, JS, ML, JN, LJ, JM, GE, and BG are employees of Pfizer and own Pfizer stock. The other authors (GQ, GN, RC, JK, AM, MC, NM, JD) have no relevant conflicts of interest to declare. The AvonCAP study is a University of Bristol sponsored study which is investigator-led, and funded under a collaborative agreement by Pfizer Inc.'

2. Please ensure that the title includes the study design.

Done – the title is (line 1):

'Impact of SARS-CoV-2 infective exacerbation of chronic obstructive pulmonary disease (COPD) on clinical outcomes in a prospective cohort study of hospitalised adults.'

3. Please ensure that the abstract is structured according to the style of JRSM research papers (see JRSM or JRSM Short Reports website). It should be no longer than 250 words.

Done – the abstract is 250 words long and structured by sections: Objectives, Design, Setting, Participants, Main outcome measures, Results, Conclusions.

4. Please structure your discussion according to this editorial http://bmj.bmjjournals.com/cgi/content/full/318/7193/1224.

Done – we have restructured the discussion to comply with this structure.

5. Please provide full details of ethical approval.

Done – we include an ethical approval statement in the manuscript (line 179):

'The study was approved by the Health Research Authority Research Ethics Committee East of England, Essex, reference 20/EE/0157. Informed consent was obtained from cognisant patients, and declarations for participation from consultees for individuals lacking capacity. Patients who declined consent were not included in this analysis. If it was not practical to approach individuals for

consent, data were included using approval from the Clinical Advisory Group under Section 251 of the 2006 NHS Act.'

6. Please provide full details of funding and sponsorship of the study.

Done – we include a funding statement (line 490):

'AvonCAP is a University of Bristol sponsored study which is investigator-led and funded under a collaborative agreement by Pfizer Inc. The study funder had no role in data collection, but collaborated in study design, data interpretation and analysis, and writing this manuscript. The corresponding author had full access to all study data and had final responsibility for the publication decision.'

7. Please nominate an author as a guarantor for the work. The guarantor accepts full responsibility for the work and/or the conduct of the study, had access to the data, and controlled the decision to publish. See http://bmj.com/cgi/content/full/323/7313/588.

Thank you – we nominate Dr Catherine Hyams (GMC 7169721) as the guarantor for this work.

8. Please provide a contributorship statement. See http://www.bmj.com/cgi/content/full/315/7110/696 and http://www.jrsm.org/cgi/content/full/99/11/542.

Done – we include a funding statement (line 501)

'CH, GQ, RC, EB, JS, JN, JO, GE, JMM, BG, LD, AF and JD generated the research questions and analysis plans. The AvonCAP research team, CH, JK, AM, and MC were involved in data collection. CH, MC and AF verified the data. CH, GQ, GN, RC, EB, JS, JN, GE, JMM, NM, AF, LD and JD undertook data analysis. All authors contributed to preparation of the manuscript and its revisions before publication. JD, BG and AF provided oversight of the research.'

9. Please report according to CONSORT/QUORUM for an RCT or systematic review, although you do need to conform to the JRSM style for abstracts and titles. Provide CONSORT/QUORUM statements and flowcharts as required.

Done – we have provided a CONSORT statement

10. Your paper should be no longer than 3000 words and 30 references.

Done – the word count is 2998 and we have 30 references.

11. Please provide a covering letter explaining how you have addressed all the points raised by the reviewer and by me.

Done – this response includes a covering letter and is a point-by-point response to you and the reviewer.

12. Please submit the revision as quickly as possible to expedite publication. If the revision takes longer than 2 months I may have to send the paper for review again.

Done
